# Nuclear GRP75 Binds Retinoic Acid Receptors to Promote Neuronal Differentiation of Neuroblastoma

**DOI:** 10.1371/journal.pone.0026236

**Published:** 2011-10-14

**Authors:** Yu-Yin Shih, Hsinyu Lee, Akira Nakagawara, Hseuh-Fen Juan, Yung-Ming Jeng, Yeou-Guang Tsay, Dong-Tsamn Lin, Fon-Jou Hsieh, Chien-Yuan Pan, Wen-Ming Hsu, Yung-Feng Liao

**Affiliations:** 1 Laboratory of Molecular Neurobiology, Institute of Cellular and Organismic Biology, Academia Sinica, Taipei, Taiwan; 2 Institutes of Zoology, National Taiwan University, Taipei, Taiwan; 3 Department of Life Science, National Taiwan University, Taipei, Taiwan; 4 Department of Molecular Biology and Oncology, Chiba Cancer Center Research Institute, Chiba University Graduate School of Medicine, Chiba, Japan; 5 Institute of Molecular and Cellular Biology, National Taiwan University, Taipei, Taiwan; 6 Department of Pathology, National Taiwan University Hospital and National Taiwan University College of Medicine, Taipei, Taiwan; 7 Institute of Biochemistry and Molecular Biology and Proteomics Research Center, National Yang-Ming University, Taipei, Taiwan; 8 Department of Pediatrics, National Taiwan University Hospital and National Taiwan University College of Medicine, Taipei, Taiwan; 9 Childhood Cancer Foundation, Taipei, Taiwan; 10 Graduate Institute of Clinical Medicine, National Taiwan University College of Medicine, Taipei, Taiwan; 11 Department of Surgery, National Taiwan University Hospital and National Taiwan University College of Medicine, Taipei, Taiwan; Seattle Children's Research Institute, United States of America

## Abstract

Retinoic acid (RA) has been approved for the differentiation therapy of neuroblastoma (NB). Previous work revealed a correlation between glucose-regulated protein 75 (GRP75) and the RA-elicited neuronal differentiation of NB cells. The present study further demonstrated that GRP75 translocates into the nucleus and physically interacts with retinoid receptors (RARα and RXRα) to augment RA-elicited neuronal differentiation. GRP75 was required for RARα/RXRα-mediated transcriptional regulation and was shown to reduce the proteasome-mediated degradation of RARα/RXRαin a RA-dependent manner. More intriguingly, the level of GRP75/RARα/RXRα tripartite complexes was tightly associated with the RA-induced suppression of tumor growth in animals and the histological grade of differentiation in human NB tumors. The formation of GRP75/RARα/RXRα complexes was intimately correlated with a normal MYCN copy number of NB tumors, possibly implicating a favorable prognosis of NB tumors. The present findings reveal a novel function of nucleus-localized GRP75 in actively promoting neuronal differentiation, delineating the mode of action for the differentiation therapy of NB by RA.

## Introduction

Neuroblastoma (NB) is the most common and deadly cancer in patients who are identified during the first year of life, and are often diagnosed as an aggressive and metastatic disease that leads to high mortality [1]. Despite the noted improvement in the overall outcome in patients with NB, the 5-year survival rates among children with high-risk NB have only improved slightly. This result is mainly attributed to the fact that key molecular pathways controlling NB tumorigenesis remain elusive.

Current treatments for NB include a combination of chemotherapy, surgery, radiation, bone marrow transplantation, immunotherapy, and differentiating agents [2]. NB is the only pediatric cancer treated with differentiation reagents as the first-line of defense [2]. One of the most potent differentiation inducers for NB is retinoic acid (RA) [3], [4]. The results of independent trials have consistently shown that the administration of RA significantly improves the overall survival rates after bone-marrow transplantation [5], [6], [7]. Consistent with these findings, NB cells treated with all-trans RA at doses used clinically display evident neuronal differentiation and reduced proliferation [8]. Therefore, the elucidation of molecular mechanisms underlying RA-induced neuronal differentiation in NB could pave the way for the development of novel therapeutic strategies for NB.

RA-elicited signaling is primarily mediated by retinoid receptors [9]. The retinoid receptors can be categorized into two subfamilies: retinoic acid receptors (RARs) and retinoid X receptors (RXRs). Upon binding RA, the ligand-bound RAR/RXR heterodimers undergo a conformational change, causing their translocation to the nucleus, where they act as transcription factors targeting retinoic acid responsive elements (RARE) within the promoters of genes involved in differentiation and growth arrest [10], [11]. Recent studies show that the activity of RA receptors can be regulated by posttranslational modifications, such as phosphorylation and ubiquitination [12]. Moreover, the ligand-induced transactivation of RAR/RXR heterodimers could also be modulated by various adaptor proteins in the nucleus [13].

Glucose-regulated protein 75 (GRP75) was first identified as a member of the Hsp70 family that could function in multiple subcellular compartments [14]. Accumulated evidence has demonstrated the versatility of GRP75 in regulating cellular stress responses, mitochondrial homeostasis, intracellular trafficking, antigen presenting, cell proliferation, differentiation, and tumorigenesis [15]. Previous work from our group demonstrated that GRP75 is upregulated in RA-treated NB cells and in NB patients with favorable prognostic outcome [16]. The present study further investigates the possible role of altered GRP75 expression in the regulation of RA-elicited neuronal differentiation in NB.

## Results

### Nuclear translocation of GRP75 is significantly enhanced upon retinoic acid-induced neuronal differentiation in neuroblastoma cells

We previously demonstrated that GRP75 expression is significantly increased in retinoic acid-treated NB cells and is associated with a favorable prognosis in NB patients [16]. In the present study, immunofluorescence confocal microscopy revealed that GRP75 was highly enriched in the nuclei of RA-treated NB cells compared to untreated cells. The cross-sectional views of stacked images unambiguously revealed that the nuclear translocation of GRP75 was significantly enhanced for an approximately 4-fold increase in RA-treated cells compared to DMSO-treated controls (Figure S1A-C). To further confirm the RA-induced nuclear localization of GRP75, the levels of GRP75 in both nuclear and cytoplasmic fractions prepared from RA- and DMSO-treated NB cells were determined by Western blotting, and the results showed a dramatic increase in nuclear GRP75 in RA-treated cells compared to control cells, while the cytosolic pools of GRP75 remained unchanged in response to RA treatment (Figure S1D). We also found that RA can induce the nuclear translocation of GRP75 in a dose-dependent manner (Figure S13). This RA-induced nuclear translocation of GRP75 was independently confirmed in two separate NB cell lines (SK-N-DZ and SK-N-SH NB, Figure S12). Together, the present findings provide the first direct evidence that GRP75 can be localized to the nucleus, where it potentially plays a critical role in the RA-elicited neuronal differentiation of NB cells.

### The interaction between nuclear GRP75 with retinoic acid receptors is significantly enhanced in differentiated NB cells

**Figure 1:**
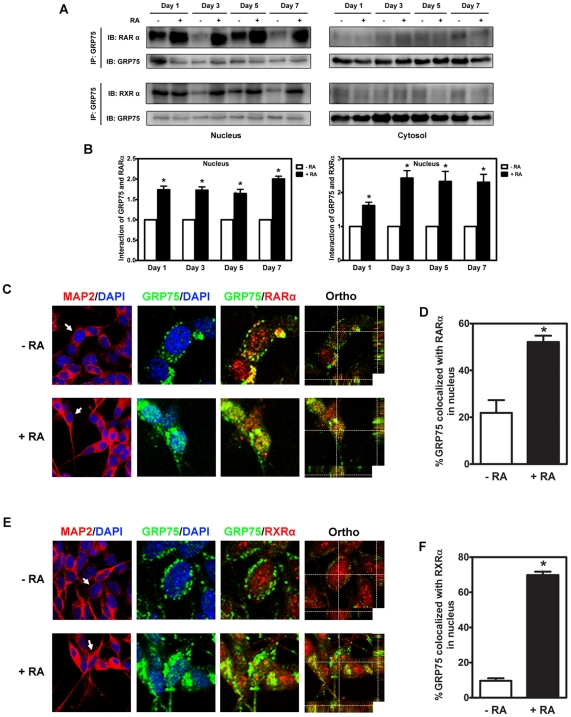
Nuclear GRP75 physically interacts with RARα/RXRα in differentiated NB cells. (A and B) Nuclear and cytoplasmic extracts were prepared from SH-SY5Y cells treated with RA (10 µM) or vehicle alone (0.1% DMSO) for various intervals. Clarified lysates of treated cells were subjected to immunoprecipitation using anti-GRP75 antibodies, followed by Western blot analysis with anti-RARα or RXRα. The levels of immunoprecipitated nuclear RARα and RXRα were normalized with those of nuclear GRP75 from the same immunopreciptate. The ratio of RARα or RXRα to GRP75 in DMSO-treated cells at a specific interval was referred to as 1 fold of relative interaction. Quantitative results are shown as the mean interactions of GRP75 with RARα or RXRα (±SEM) of three individual experiments and were analyzed by Student's t test. *p<0.05. (C-F) Immunofluorescence staining representing the colocalization between GRP75 and RARα/RXRα. SH-SY5Y cells were treated with RA (10 µM) or vehicle alone (0.1% DMSO) for 3 d and processed for immunofluorescence staining with anti-MAP2, anti-GRP75, and either anti-RARα or RXRα. Nuclei were visualized by DAPI counterstaining. Three-dimensional analysis of the co-localization of GRP75 and either RARα or RXRα by *z*-stack images was denoted by intersecting lines in the *x*, *y*, and *z* axes. Scale bar = 20 µm. Overlapped pixels (yellow) corresponding to GRP75 (green) and either RARα (C, red) or RXRα (E, red) were defined as the co-localization of GRP75 with either RARα or RXRα. The co-localization of GRP75 with either RARα (D) or RXRα (F) was determined as the mean (±SEM) percentages of nuclear GRP75-specific pixels overlapped with either RARα- or RXRα-specific pixels from at least three different viewing areas per experiment in three independent experiments and analyzed by Student's t test. *p<0.05.

RA primarily binds to RARα/RXRα heterodimers to transduce its downstream signaling [17]. It is therefore possible that the nucleus-localized GRP75 could be actively involved in the RA-induced neuronal differentiation of NB cells by interacting with RA receptors and modulating their activities. To investigate this possible mechanism, the potential association between GRP75 and RARα/RXRα in the nucleus in response to RA-induced neuronal differentiation was investigated. Using co-immunoprecipitation, a physical interaction between endogenous GRP75 and RARα and RXRα in the nucleus was demonstrated. This novel interaction between GRP75 and RARα/RXRα was significantly enhanced in the nucleus in RA-treated NB cells compared to controls, but it was trivial and irresponsive to RA treatment in the cytosol (Figure 1A, B). Co-immunoprecipitation with a non-specific mouse IgG did not reveal any detectable interaction between GRP75 and RA receptors (Figure S11). Moreover, the co-localization of GRP75 with RARα/RXRα heterodimers was vividly observed in the nucleus of RA-treated SH-SY5Y cells and increased upon RA stimulation (Figure 1C-F, Figure S2), suggesting that RA could selectively and significantly enhance the binding of GRP75 to RARα/RXRα. The interaction between GRP75 and RARα/RXRα was independently validated in two *MYCN*-nonamplified (SK-N-SH and SK-N-MC) and three *MYCN*-amplified NB cell lines (SK-N-DZ, SK-N-BE, and IMR-32) (Figure S3), suggesting a critical role of GRP75 in RA signaling and NB differentiation. Together, these results strongly suggest that GRP75 participates in RA-elicited neuronal differentiation of NB cells through its direct interaction with nuclear RARα/RXRα heterodimers.

### Down-regulation of GRP75 inhibits RA-elicited activation of RARα/RXRα receptors

**Figure 2:**
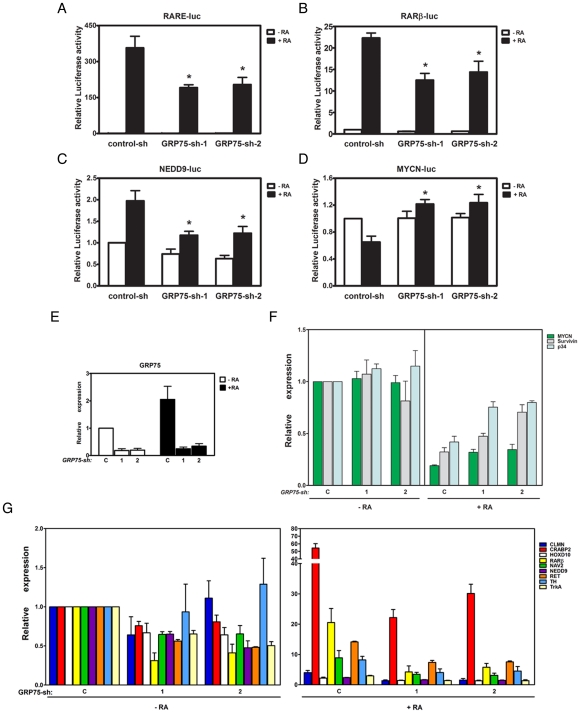
GRP75 is required for the transcriptional regulation of RA target genes in NB cells. (A–D) SH-SY5Y cells were infected with lentivirus encoding shRNA targeting GFP (Control-sh) or GRP75 (GRP75-sh-1 and -2) for 2 d, and infected cells were then transfected with RARE-Luc (A), *RARβ* promoter-Luc (B), NEDD9-Luc (C), or the MYCN-Luc (D) reporter gene construct for an additional 2 d. Following treatment with RA (10 µM) or vehicle alone (0.1% DMSO) for 24 h at 37°C, the luciferase signals in clarified lysates of treated cells were determined and normalized to protein concentration. Normalized luciferase signal of DMSO-treated Control-sh-infected cells were referred to as one fold of relative luciferase activity. (E) shRNA-infected SH-SY5Y cells treated with RA or DMSO as described above were harvested and processed for total RNA isolation by TRIzol reagent. The levels of GRP75 mRNA transcript as well as GAPDH (internal control) were determined by real-time RT-PCR. The normalized level of GRP75 transcript in Control-sh-infected DMSO-treated cells was referred to as 1 fold of relative GRP75 expression. Quantitative data were calculated as the mean (±SEM) relative GRP75 expression of triplicate measurements from three independent experiments and analyzed by Student's t test. (F-G) Total RNA transcripts of shRNA-infected SH-SY5Y cells treated with RA or DMSO were analyzed by real-time RT-PCR for the expression of RA target genes essential for cell proliferation (F) and neuronal differentiation (G). All quantitative data were calculated as the mean (±SEM) from three independent experiments and analyzed by Student's t test. *p<0.05.

To determine whether the binding of GRP75 to the RARα/RXRα receptor complex in the nucleus is necessary for the RA-induced neuronal differentiation of NB cells, the expression of RA target genes was examined in GRP75-deficient SH-SY5Y cells in the presence or absence of RA. Using an RARα/RXRα-driven luciferase reporter gene construct (RARE-Luc), RA-induced reporter gene expression was determined to be abrogated by the down-regulation of GRP75, suggesting that GRP75 is required for RA-triggered transcriptional regulation (Figure 2A). This finding was further corroborated by data showing that GRP75-deficient SH-SY5Y cells exhibit a significant reduction in the RA-elicited activation of the *RARβ* promoter, a known RARα/RXRα downstream target gene [18] (Figure 2B). The knockdown efficiency of both GRP75-targeting shRNAs was approximately 75%, as seen in the mRNA transcript and protein levels (Figure 2E and Figure S4). Using reporter gene constructs derived from MYCN and NEDD9 promoters [19], [20], the depletion of GRP75 in SH-SY5Y cells was further demonstrated to effectively inhibit the RA-elicited stimulation of NEDD9 promoter activity (a neuronal differentiation marker) and abolish the RA-triggered suppression of MYCN promoter activity (a proliferation marker) (Figures 2C, D). The present data clearly suggest that GRP75 could be a novel transcriptional coactivator of RARα/RXRα and therefore modulate RA-elicited neuronal differentiation of NB cells.

To further substantiate the role of GRP75 in RARα/RXRα-mediated transcriptional regulation, the mRNA transcript levels of selected RA target genes were examined by real-time quantitative PCR. Several RA target genes, such as CLMN, CRABP2, HOXD10, RARβ, NAV2, NEDD9, RET, TH, and TrkA, have been shown to promote neuronal differentiation, whereas others, including MYCN, Survivin, and p34, function in cell proliferation [19], [21], [22], [23], [24]. In the present study, while the expression of nine RA target genes involved in neuronal differentiation was significantly elevated in RA-treated NB cells compared to controls, knockdown of GRP75 dramatically compromised the RA-induced transcriptional up-regulation of these pro-differentiation target genes in NB cells (Figure 2G). Concomitant with defective neuronal differentiation, down-regulation of GRP75 could favor cell growth by diminishing the RA-elicited transcriptional suppression of pro-proliferation target genes (MYCN, Survivin, and p34) (Figure 2F).

**Figure 3:**
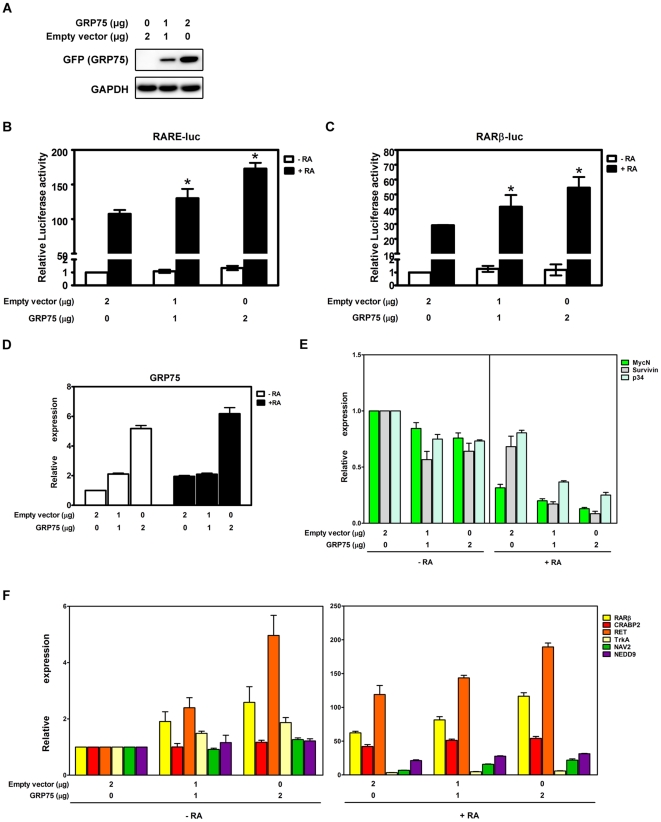
Overexpression of GRP75 augments RA-elicited activation of RARα/RXRα-mediated transcriptional regulation in SH-SY5Y cells. (A) SH-SY5Y cells were transiently transfected with an empty vector or a GRP75-expression vector for 24 h. Ectopic expression of GFP-GRP75 in RA-treated transfected cells was analyzed by Western blot analysis with anti-GFP antibody (upper panel, for GRP75). GAPDH (lower panel) was used as a protein loading control. (B and C) SH-SY5Y cells were transiently co-transfected with a RA-responsive reporter gene construct (2 µg of RARE-Luc or RARβ-Luc) and a GRP75-expressing construct at various concentrations for 24 h, followed by treatment with 10 µM RA for 24 h. Luciferase signals derived from reporter gene constructs were determined by Steady-Glo luciferase assay reagents and normalized by protein concentration. The normalized luciferase signal in DMSO-treated cells transfected with an empty vector alone was referred to as 1 fold of relative luciferase activity. Quantitative results are presented as the mean (±SEM) of triplicate measurements from three independent experiments and were analyzed by Student's t test. *p<0.05. (D) The levels of GRP75 mRNA transcripts in SH-SY5Y cells transiently transfected with a GRP75-expressing vector were determined by quantitative real-time RT-PCR. The normalized level of GRP75 transcripts in DMSO-treated cells transfected with an empty vector alone was referred to as 1 fold of relative expression. Quantitative results are presented as the mean (±SEM) of triplicate measurements from three independent experiments. (E and F) SH-SY5Y cells were transfected with an empty vector or a GRP75-expressing vector for 48 h, followed by treatment with or without RA for 24 h. The transcript levels of various RA-responsive genes in transfected cells were determined by quantitative real-time RT-PCR. The normalized transcript level in DMSO-treated cells transfected with an empty vector alone was referred to as 1 fold of relative expression. Quantitative results are presented as the mean (±SEM) of triplicate measurements from three independent experiments and were analyzed by Student's t test.

Consistent with the essential role of GRP75 in neuronal differentiation, the ectopic expression of GRP75 in SH-SY5Y cells significantly enhanced RA-triggered transactivation of RARα/RXRα (Figure 3A-C). The GRP75-dependent transcriptional regulation of RA target genes was further explored through the ectopic expression of GRP75 in SH-SY5Y cells. Overexpression of GRP75 significantly potentiated the expression of differentiation-promoting genes and suppressed the expression of pro-proliferation genes in NB cells with or without RA (Figure 3E, F). Together, the regulation of RA-elicited neuronal differentiation by GRP75 was independently confirmed in separate albeit related NB cell lines SH-SY5Y and SK-N-SH (Figure 2, Figure 3, Figure S9, and Figure S10). The present results clearly demonstrate for the first time that GRP75 can be actively involved in RARα/RXRα-mediated transcriptional regulation in RA-triggered neuronal differentiation of NB cells.

### GRP75/RARα/RXRα complexes cooperatively bind to the retinoic acid response element (RARE) within the promoters of RA-responsive genes

**Figure 4:**
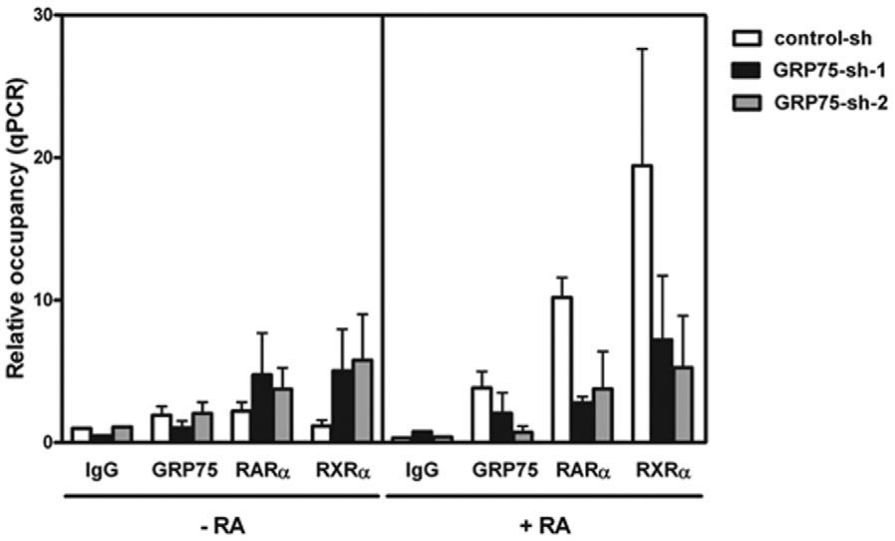
GRP75 is an obligatory constituent of RARα/RXRα-containing transcription machinery. (A) Down-regulation of GRP75 reduces the RA-elicited binding of RARα/RXRα heterodimers to the RARE motif. SH-SY5Y cells were infected by a lentivirus expressing control shRNA (Control-sh) or GRP75-targeting shRNAs (GRP75-sh-1 and -2) for 2 d. Infected cells were grown in the presence or absence of RA (10 µM) for 24 h. Soluble chromatins of treated cells were cross-linked by formaldehyde and sonicated to shear DNA. Protein-bound chromatin fragments were immunoprecipitated using a control mouse IgG antibody, mouse anti-GRP75, rabbit anti-RARα, or rabbit anti-RXRα antibody. The levels of DR5 RARE sequences within the RARβ promoter eluted from antibody-reactive immunoprecipitates were determined by quantitative real-time PCR. Sonicated total DNA fragments were used as the input DNA. The level of DR5 RARE normalized with input DNA in control IgG-reactive immunoprecipitates derived from DMSO-treated Control-sh-infected cells was referred to as one fold of relative occupancy. The results are shown as the mean (±SEM) from three independent experiments and were analyzed by Student's t test. p<0.05.

To further demonstrate the functional role of the GRP75-bound RARα/RXRα receptor complex in RA-elicited transcriptional regulation, the RARα/RXRα-mediated binding to DR5 RARE within the promoter of *RARβ*, a direct target gene downstream of RA-elicited signaling [18], was examined using chromatin immunoprecipitation (ChIP) in SH-SY5Y cells infected with lentiviral particles expressing shRNAs targeting GFP (Control-sh) or GRP75 (GRP75-sh-1 and GRP75-sh-2) in the presence or absence of RA. The RNAi-mediated down-regulation of GRP75 significantly diminished the recruitment of RARα and RXRα receptors to RARE-containing promoter regions in RA-treated NB cells (Figure 4A), suggesting that GRP75 can be recruited to RARE consensus sequence within the promoter of *RARβ* and indispensable for the binding of RARα/RXRα receptor complexes to the promoters of RA target genes and that it critically modulates the RA-elicited expression of pro-differentiation genes. Since GRP75, RARα, and RXRα are all indispensable for cell viability, the depletion of GRP75 in SH-SY5Y cells in the absence of RA could compromise the basal viability of NB cells [9], [25], [26]. It is thus possible that the recruitment of RARα/RXRα to the RARE region could be enhanced to compensate the loss in GRP75-dependent maintenance of cell viability in response to GRP75 knockdown. These findings thus favor a model in which the binding of GRP75 is a prerequisite for the efficient association of RARα/RXRα complexes with RARE-containing promoter regions to modulate the expression of RA target genes in the regulation of neuronal differentiation.

### Down-regulation of GRP75 promotes RARα/RXRα degradation through a proteasome-mediated pathway

**Figure 5:**
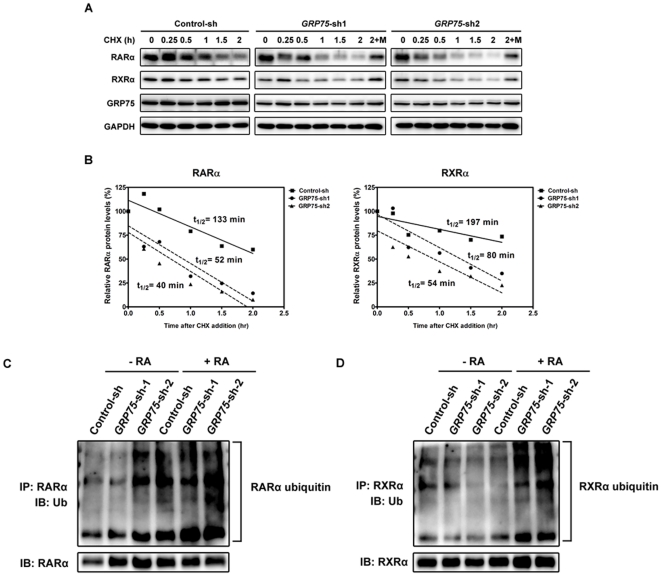
Deficiency in GRP75 promotes the degradation of RARα/RXRα heterodimers by the ubiquitin proteasome system. (A and B) SH-SY5Y cells were infected with a control shRNA (Control-sh) or GRP75-specific shRNAs (GRP75-sh-1 and -2) for 2 d. Cells were then treated with 50 µg/ml of cycloheximide (CHX) and 10 µM RA for various intervals. The protein levels of RARα, RXRα, GRP75, and GAPDH (protein load control) were analyzed by SDS-PAGE and immunoblotting (A). The relative levels of RARα (left panel) and RXRα (right panel) in infected cells prior to being treated with cycloheximide and RA were referred to as 100% (B). (C and D) SH-SY5Y cells infected with gene-targeting lentiviral shRNAs (Control-sh, GRP75-sh-1, and GRP75-sh-2) were treated with MG-132 (5 µM) in the presence or absence of RA (10 µM) for 16 h. Clarified lysates were subjected to immunoprecipitation with either anti-RARα or anti-RXRα. The ubiquitinated RARα (C) and RXRα (D) in immunoprecipitates were resolved by SDS-PAGE and visualized by immunoblotting with an anti-ubiquitin antibody. The same blots were reprobed with anti-RARα or anti-RXRα to reveal the input levels of both receptors (lower panel).

Accumulated evidence also suggests that the ubiquitin proteasome system (UPS)-mediated degradation and the binding to molecular chaperones can modulate the activity of steroid receptors, including RAR/RXR retinoid receptor family [27], [28], [29], [30]. We thus demonstrated that RNAi-mediated down-regulation of GRP75 in RA-treated SH-SY5Y cells resulted in a significant increase in the ubiquitination and UPS-mediated degradation of ligand-bound RARα/RXRα, compared to mock-infected cells (Figure 5). Consistent with its protective role, the overexpression of GRP75 resulted in a significant attenuation in the ubiquitination of both RARα and RXRα (Figure S5). Together, these findings suggest that GRP75-dependent increase in the stability of ligand-bound RARα/RXRα heterodimers could selectively potentiate ligand-bound RARα/RXRα-mediated transcriptional regulation, synergistically augmenting RA-elicited neuronal differentiation.

### An enhanced interaction between GRP75 and RARα/RXRα heterodimers is associated with favorable outcomes in an in vivo xenograft NB mouse model

**Figure 6:**
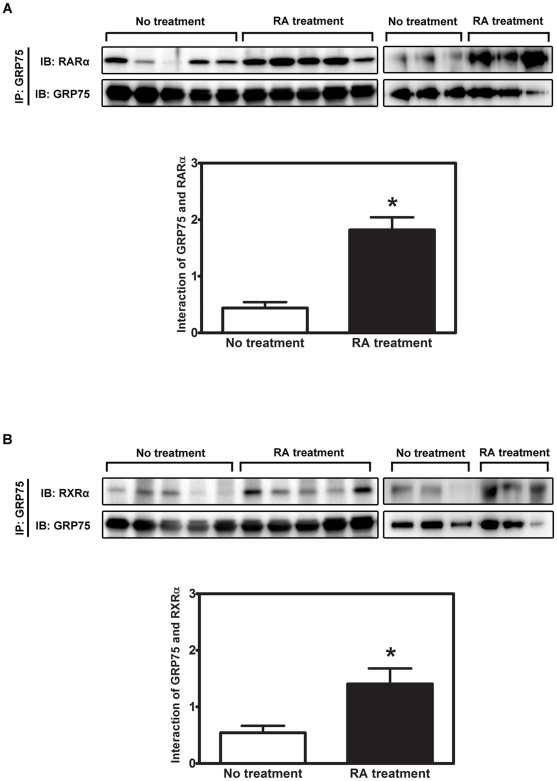
The interaction between GRP75 and RARα/RXRα is significantly increased in mouse NB xenografts treated with RA. Male nude mice were subcutaneously inoculated with 1×10^7^ stNB-V1 NB cells with matrigel. Once the size of the xenograft tumor reached approximately 1 cm^3^, tumor-bearing mice were intraperitoneally treated with RA (1 mg/kg b.w.) or vehicle (0.1% DMSO) daily for 14 d. Clarified lysates derived from xenograft tumors were subjected to immunoprecipitation with an anti-GRP75 antibody. Immunoprecipitated proteins were analyzed by SDS-PAGE and immunoblotting with anti-RARα (A) or RXRα (B). The same blots were reprobed with anti-GRP75 to reveal the input level of GRP75 (lower panel). The ratios of RARα to GRP75 (A) and RXRα to GRP75 (B) were referred to as the interaction between GRP75 and respective receptors. Quantitative results are shown as the mean (±SEM) of 8 xenografts from two independent experiments and were analyzed by Student's t test. *p<0.05.

To validate the significance of the interaction between GRP75 and RARα/RXRα heterodimers in the modulation of RA-elicited neuronal differentiation *in vivo*, the correlation of these novel protein-protein interactions with tumor progression was examined in a xenograft NB mouse model [31]. We found that RA treatment significantly inhibits tumor growth beginning on the fourth treatment day until the completion of the treatment period in comparison to vehicle-treated control animals based on tumor size measurements. The assessment of harvested tumor xenografts at the conclusion of treatment revealed that both tumor size and tumor weight were significantly decreased in RA-treated mice (Tables S1), indicating that RA therapy can effectively inhibit the progression of NB. The correlation between the level of GRP75/RARα/RXRα tripartite complexes and tumor growth was further analyzed in the harvested NB xenografts. Our data showed that the association of GRP75 with either RARα or RXRα is significantly increased in RA-treated xenografts compared to controls (Figure 6, Table S1). Linear correlation analysis demonstrated that the formation of either GRP75/RARα or GRP75/RXRα complexes was markedly elevated in tumors with reduced volume or weight (RA-treated xenografts), in stark contrast to the control tumors with larger volume or weight (Figure S6). These results clearly demonstrate that the complex formation of tripartite GRP75/RARα/RXRα is inversely correlated with the progression of NB.

### The interaction between GRP75 and RXRα/RARαis correlated with a higher grade of histological differentiation and a normal MYCN copy number in human NB tumors

**Figure 7:**
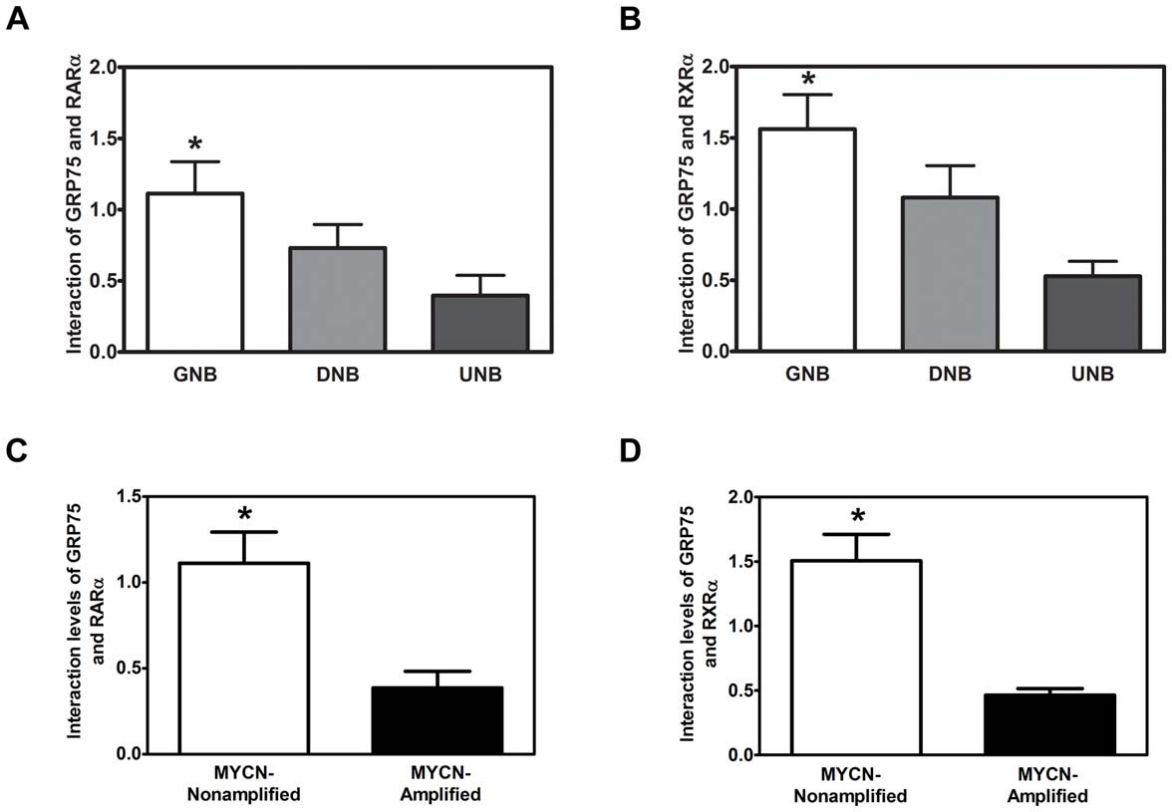
The interaction between GRP75 and RARα/RXRα in primary NB tumors is tightly associated with the differentiation histology but is inversely correlated with MYCN amplification. (A and B) Tumors collected from NB patients with different histology statuses, including ganglioneuroblastoma (GNB), differentiated neuroblastoma (DNB), and undifferentiated neuroblastoma (UNB), were processed for immunoprecipitation with anti-GRP75. The levels of GRP75-interacting RARα and RXRα in immunoprecipitates were analyzed by immunoblotting with anti-RARα (A) or anti-RXRα (B). The same blots were reprobed with anti-GRP75 to reveal the input level of GRP75. The ratios of RARα to GRP75 (A) and RXRα to GRP75 (B) were referred to as the interaction between GRP75 and the respective receptors. Quantitative results are shown as the mean (±SEM) interaction between GRP75 and RARα (A) or RXRα (B) and were analyzed by Student's t test. *p<0.05. The images of Western blots for the GRP75 immunoprecipitation used for this analysis wwer included in the Figure S8. Human NBs were classified by histology based on the criteria established by the International Neuroblastoma Pathology Classification [53]. GNB (n = 13); DNB (n = 9); UNB (n = 8). (C and D) The correlation between the levels of GRP75/RARα/RXRα complexes and MYCN status in primary NB tumors from the same cohort was determined. Quantitative data are shown as the mean (±SEM) interaction between GRP75 and RARα (C) or RXRα (D) in tumors without MYCN amplification (MYCN-nonamplified, n = 16) or those with MYCN amplification (MYCN-amplified, n = 5) and were analyzed by Student's t test. *p<0.05.

To corroborate the relationship between the RA-induced formation of nucleus-localized GRP75/RARα/RXRα tripartite complexes with the differentiation of human NB, the interaction between GRP75 and RARα/RXRα heterodimers was examined in NB tumors with various grades of histological differentiation. Using co-immunoprecipitation with an anti-GRP75 antibody and lysates derived from 30 human NB tumors, including 13 ganglioneuroblastomas (GNBs), 9 differentiating NBs (DNBs), and 8 undifferentiated NBs (UNBs), the interaction between GRP75 and RARα/RXRα was found to be significantly stronger in tumors with higher grades of histological differentiation (GNB and DNB) than in those with an undifferentiated histology (UNB) (Figure 7A, B and Figure S8). Furthermore, the interaction between GRP75 and RARα/RXRα was higher in tumors with a normal MYCN copy number compared with those with MYCN amplification which carry a very unfavorable prognosis (Figure 7C, D). These pieces of evidence were consistent with our finding that GRP75 is required for RA-elicited down-regulation of MYCN expression (Figure 2D, F). The present data thus unequivocally support a model in which the level of tripartite GRP75/RARα/RXRα complexes in NB tumors is tightly associated with the histological grade of differentiation and, possibly, a favorable prognosis of NB tumors. Our data thus favors a model in which, upon RA-induced neuronal differentiation, GRP75 could be recruited to the ligand-bound RARα/RXRα heterodimers to cooperatively regulate the expression of RA downstream genes and avert UPS-mediated degradation of RA-bound RARα/RXRα (Figure S7A). Given that the molecular structure of human GRP75 has not been resolved, we then based on the structure of *Escherichia coli* HSP70 chaperone chain A (PubMed accession P0A6Y8, Protein Data Bank code 2KHO_A) that exhibits the highest amino acid sequence identity to human GRP75 to predict the three-dimensional model of GRP75. We then further simulated the possible docking of modeled GRP75 structure to known RARα or RXRα structure. This molecular simulation predicted that GRP75 could bind to the ligand-binding domain or the DNA-binding domain of RARα/RXRα heterodimers (Figure S7B, C). In conclusion, the present findings further strengthen the prognostic value of the expression level of GRP75 in NB [16], and delineate the mechanism underlying the GRP75-dependent regulation of RA-elicited RARα/RXRα-dependent neuronal differentiation.

## Discussion

GRP75 has been shown to bind multiple partner proteins to govern diverse cellular functions [14], [32]. The alteration in the cellular distribution of GRP75 could also correlate with the status of cellular immortality [33], [34]. Consistent with this notion, our data clearly show that the association of GRP75 with RARα and RXRα is remarkably increased in the nucleus and coincides with the RA-elicited growth arrest, concomitant with a tight correlation between RA-induced nuclear translocation of GRP75 and RA-triggered neuronal differentiation. These data strongly favor a model in which nuclear GRP75 could stably form complexes with RARα/RXRα heterodimers and actively participate in RA-triggered neuronal differentiation of NB cells through the persistent modulation of RARα/RXRα activity (Figure S7).

The present data provide the first direct evidence that nucleus-localized GRP75 is essential for RARα/RXRα-mediated transcriptional regulation and that the GRP75/RARα/RXRα tripartite complexes physically bind to RARE to modulate the expression of RA target genes for neuronal differentiation (Figures 2, 3, 4). These results also support previous findings showing that RA-elicited down-regulation of MYCN expression is a prerequisite for the neuronal differentiation of NB cells, while constitutive overexpression of MYCN can counteract RA-induced neuronal differentiation [35], [36]. GRP75 could thus play a central role in coordinating the transcriptional regulation of MYCN expression through the stimulation of RARα/RXRα activity, modulating a positive auto-regulatory loop for MYCN in NB cells [20]. GRP75 could also act as a transcriptional co-activator to potentiate RARα/RXRα-mediated transactivation of pro-differentiation genes, constituting a positive feedback loop that can potentiate RA-induced neuronal differentiation of NB cells. Consistent with the essential role of GRP75 in neuronal function [37], our results thus strengthen the notion that the functions of retinoid receptors, like other nuclear receptors, could be regulated by molecular chaperones, such as GRP75, in an evolutionarily conserved fashion [38], [39], [40].

The activity of nuclear receptors could be regulated by post-translational modifications, including phosphorylation and ubiquitination [41]. Previous studies have shown that phosphorylation of RARs within their TFIIH binding sites is essential for RA-mediated embryonic development and underlies the pathogenesis of xeroderma pigmentosum [42]. RARs have also been shown to interact with SUG-1 to induce its ubiquitination and degradation by the proteasome upon RA stimulation [43]. A recent study revealed that Hsp27 can form a complex with androgen receptor (AR) and co-migrate into the nucleus to modulate the transcriptional activity of AR through an alteration in the UPS-mediated degradation of AR [28]. The activity of RA receptors was also shown to be modulated by interacting proteins, such as calreticulin [44]. Consistent with the functional roles of chaperones [45], the present results showing that GRP75 interacts with RARα and RXRα and is involved in the UPS-mediated degradation of these receptors further substantiate the idea that the magnitude and function of retinoid-elicited signaling could depend on the efficiency of the UPS-mediated processing of ligand-bound retinoid receptors (Figure 5 and Figure S5). A recent study showed that the molecular chaperone HSP90 binds to the E3 ubiquitin ligase CHIP and prevents CHIP-mediated degradation of leucine-rich repeat kinase 2 (LRRK2) [46]. The present findings that GRP75 could stabilize the RARα/RXRα complex by modulating the UPS pathway to potentiate the RA-elicited neuronal differentiation of NB cells further support a critical role of the UPS pathway in the regulation of the activity of RA receptors [47].

The association between GRP75 and RARα/RXRα heterodimers is of clinical significance. In a xenograft mouse model of NB and a cohort of patient samples, the formation of tripartite GRP75/RARα/RXRα complexes was inversely correlated with tumor progression and consistently predicted a favorable outcome (Figure 6, Figure 7, Figure S6, and Table S1). Given that the N-terminal ATP-binding domain and the C-terminal substrate-binding domain of GRP75 are highly identical to those of human Hsp70 [48], [49], GRP75 could act like Hsp70 and modulate the conformations of RARα and RXRα. Based on the molecular modeling for the interaction between GRP75 and either RARα or RXRα (Figure S7), GRP75 is likely to interact with either the ligand-binding domain or the DNA-binding domain of RARα/RXRα heterodimers to synergistically stabilize the RA-bound GRP75/RARα/RXRα complexes and sustain their transcriptional activation. Synthetic molecules designed to either induce the de novo formation of GRP75/RARα/RXRα complexes or stabilize the pre-existent ones could thus have profound therapeutic implication for NB.

In summary, the present study identifies a novel function of GRP75 in regulating RA-elicited neuronal differentiation through direct interaction with RARα/RXRα heterodimers in the nucleus. The elucidation of the molecular mechanism involved in the formation of tripartite GRP75/RARα/RXRα complexes provides the basis for the development of a novel therapeutic strategy for NB that could be combined with other existing differentiating regimens to improve the overall outcomes of NB patients [50], [51]. Our data could serve as the foundation for the generation of molecules that could simultaneously prevent UPS-mediated degradation of RARα/RXRα and extend the pro-differentiation effect of GRP75/RARα/RXRα complexes.

## Materials and Methods

### Ethics Statement

The Institutional Review Board of National Taiwan University Hospital approved the complete follow-up protocols and this study. We obtained the written informed consent from all participants involved in this study. The animal study protocol used in this study was approved by the Institutional Animal Care and Use Committee of Academia Sinica (Approval Protocol ID #TMiZOOLY2007092). Fetal bovine serum was obtained from Invitrogen (Carlsbad, CA).

### Authentication of cell lines

The human NB cell lines SH-SY5Y (ATCC® CRL-2266™), SK-N-SH (ATCC® HTB-11™), SK-N-DZ (ATCC® CRL-2149™), SK-N-MC (ATCC® HTB-10™), and SK-N-BE(2) (ATCC® CRL-2271™) were obtained from American Type Culture Collection (Manassas, VA, USA) in November 2007. The human NB cell line IMR-32 (BCRC 60014) was obtained from Biosource Collection and Research Center (Hsinchu, Taiwan) in November 2007. These NB cell lines have been authenticated in our laboratory in a monthly basis. The SH-SY5Y and SK-N-SH cell lines were last authenticated by microscopic morphology check and PCR-based microplasma test in August 2011. The PCR-based microplasma testing was performed as previously described [50].

### Immunofluorescence confocal microscopy

SH-SY5Y cells were grown on coverslips and treated with 10 µM RA in DMSO or 0.1% DMSO alone for the indicated time. Immunofluorescence staining was performed as described previously [16].

### Reporter gene constructs and lentiviral shRNAs

The DR5-TK and RARβ-luciferase reporter constructs were kindly provided by Dr. Jonathan Kurie (The University of Texas M.D. Anderson Cancer Center) [52]. The Nedd9 promoter constructs were kindly provided by Dr. Margaret Clagett-Dame (University of Wisconsin-Madison) [19]. The shRNA lentiviral vectors targeting human GRP75 (GRP75-sh-1 and GRP75-sh-2) and a GFP-targeting control lentiviral vector (Control-sh) were purchased from the National RNAi Core Facility, Academia Sinica, Taiwan.

### Co-immunoprecipitation and Western blotting

The nuclear extracts and cytosolic pools of treated cells were isolated using the Nuclei EZ Prep Kit (Sigma) as described in the manufacturer's instructions. For co-immunoprecipitation, 20 µg of goat anti-GRP75 antibody was incubated with 200 µl of immobilized protein A agarose in PBS for 2 h at 4°C. Immunoprecipitated proteins were eluted by the addition of 4X sample loading buffer and boiling at 100°C for 10 min, followed by SDS-PAGE and Western blotting.

### Luciferase reporter assay

shRNA-transduced cells were transfected with a promoter-specific luciferase reporter gene construct (either RARE-luc, RARβ-luc, NEDD9-luc, or MYCN-luc) using Lipofectamine 2000 at 37°C overnight. Promoter-driven luminescence in the clarified lysates of transfected cells was determined using the Steady-Glo luciferase assay reagent (Promega) and normalized to the protein content of the lysates.

### RNA isolation, reverse transcription, and quantitative real-time PCR

Total RNA was isolated from transduced SH-SY5Y cells using the TRIzol Reagent (Invitrogen). Purified RNAs (2 µg) were reverse-transcribed to the first-strand cDNA using the SuperScript III First-strand cDNA Synthesis Kit (Invitrogen). Equivalent amounts of cDNA were used in quantitative real-time PCR using the SYBR Green I Master reagent on the LightCycler 480 Real-Time PCR System (Roche) with gene-specific primer pairs.

### Chromatin immunoprecipitation (ChIP)

Treated cells were fixed with 1% formaldehyde for 10 min at 37°C, and the reaction was terminated by the addition of 1.25 M glycine (1∶9, v/v). Fixed cells were lysed and sonicated in buffer A (1% SDS, 10 mM EDTA, 50 mM Tris, pH 8.0) to shear DNA. Clarified lysates were subject to immunoprecipitation using antibodies (10 µg) against either GRP75, RARα, RXRα, or mouse IgG (as control) at 4°C overnight with agitation, followed by incubation with protein A-conjugated agarose at 4°C for 1 h.

### Protein stability

Protein synthesis in infected cells was inhibited with 50 µg/ml cycloheximide for 3 h, followed by the addition of 10 µM RA or vehicle alone (0.1% DMSO) and incubation at 37°C for various intervals.

### Ubiquitination assay for RARα and RXRα

SH-SY5Y cells were infected with GRP75-sh-1, GRP75-sh-2, or control-sh at 37°C for 2 d, followed by the removal of infection mixture and incubation with fresh medium containing 10 µM RA or 0.1% DMSO for 16 h in the presence or absence of 5 µM MG132.

### Patients and sample preparation

A cohort of 30 histologically confirmed NB patients with complete follow-up protocols approved by the Institutional Review Board of National Taiwan University Hospital, Taipei, Taiwan, were enrolled in this study. The Institutional Review Board of National Taiwan University Hospital also approved this study, and we obtained informed written consent from all participants involved in this study. Tumor samples were obtained during surgery and immediately frozen in liquid nitrogen. The categorization of tumor biopsies was based on the International Neuroblastoma Pathology Classification scheme [53].

Additional information regarding details of individual experimental procedures can be found in the Supplemental Methods S1 submitted along with the main manuscript.

## Supporting Information

Figure S1 **GRP75 is translocated into the nucleus of differentiated neuroblastoma cells.** (A) Immunofluorescence microscopy analysis of GRP75 in the nuclei of NB cells. SH-SY5Y cells were treated with or without RA (10 µM) for 3 d and processed for immunofluorescence staining with an anti-GRP75 antibody (green). Nuclei were counterstained with DAPI (blue). Insets, two-fold magnification of highlighted cells (arrow). Scale bar = 20 µm. (B) Three-dimensional analysis of individual cells by *z*-stack confocal images at specific sites marked by intersecting lines in the *x*, *y*, and *z* axes. (C) Quantitative analysis of the intensity of cells double labeled for GRP75 and DAPI (nuclear DNA). Data are expressed as the average percentage (±SEM) of nuclear GRP75 co-localized with RARa from three independent experiments. *p<0.05. (D) The nuclear extracts of SH-SY5Y cells treated with or without RA for various intervals were resolved by SDS-PAGE and analyzed by immunoblotting with the indicated antibodies. Histone H1 and Lamin A/C were markers for nuclear extracts, while GAPDH was included as a protein loading control for cytosolic pools. The prolonged exposure for GAPDH blot (Nucleus) revealed no contamination of cytosolic proteins in the isolated nuclear extracts. Similarly, overexposure of histone H1- and lamin A/C-labeled blots (Cytosol) showed that isolated cytosolic pools were free from contamination of nuclear proteins.
(TIF)

Figure S2 **The interaction between GRP75 and RARα/RXRα is increased in RA-treated SH-SY5Y cells.** (A) SH-SY5Y cells were grown on coverslips and treated with 10 µM RA for various intervals. Cells treated with vehicle alone (0.1% DMSO) were included as controls. Treated cells were fixed with 4% paraformaldehyde and subjected to immunofluorescence staining using goat anti-GRP75 (green), mouse anti-MAP2 (red), and rabbit anti-RARα (red). Nuclei were counterstained with DAPI. The inset shows the magnification of the highlighted region (arrow). Scale bar = 20 µm. (B) The levels of nuclear GRP75 were quantified using MetaMorph Offline 7.5.1.0 Image Analysis System (Molecular Devices). Quantitative results are shown as the mean (±SEM) from three independent experiments. (C) The levels of GRP75 co-localized with RARα in the nucleus were quantified using the MetaMorph Offline 7.5.1.0 Image Analysis System (Molecular Devices). Quantitative data are shown as means (±SEM) of at least three different viewing areas from three independent experiments. All quantitative data were analyzed by Student's t test. *p<0.05 versus DMSO-treated control (– RA). (D) SH-SY5Y cells were treated with 10 µM RA for various intervals. Cells treated with vehicle alone (0.1% DMSO) were included as controls (– RA). Treated cells were fixed with 4% paraformaldehyde and subjected to immunofluorescence staining using goat anti-GRP75 (green), mouse anti-MAP2 (red), and rabbit anti-RXRα (red). Nuclei were visualized by DAPI staining. The inset shows the magnified view of the highlighted region (arrow). Scale bar = 20 µm. (E and F) The levels of nuclear GRP75 (E) and RXRα-co-localized GRP75 in the nucleus (F) were determined using the MetaMorph Offline 7.5.1.0 Image Analysis System (Molecular Devices). Quantitative results are shown as means (±SEM) from three independent experiments and were analyzed by Student's t test. *p<0.05 versus DMSO-treated control (– RA). The number of cells used in the quantitative analysis was at least 150.
(TIF)

Figure S3 **RA enhances the formation of GRP75/RARα/RXRα tripartite complexes in various NB cell lines.** MYCN-nonamplified NB cell lines, including SK-N-SH (A), SK-N-MC (B), and stNB-V1 (C), and MYCN-amplified NB cell lines, including SK-N-BE (D), SK-N-DZ (E), and IMR-32 (F), were treated with 10 µM RA or vehicle alone (0.1% DMSO) for 1 d. Nuclear lysates were immunoprecipitated with a mouse anti-GRP75 antibody. Protein A-bound antigen-antibody complexes were analyzed by immunoblotting with anti-RARα (upper panel), anti-RXRα (middle panel), or anti-GRP75 (lower panel, loading control). The levels of RARα and RXRα were normalized to GRP75 from the same immunoprecipitate, and those in cells without RA treatment were referred to as one fold of relative interaction between GRP75 and respective RA receptors (RARα and RXRα).
(TIF)

Figure S4 **Lentiviral shRNA-mediated down-regulation of GRP75 in SH-SY5Y cells.** SH-SY5Y cells were infected with lentiviral shRNA targeting GFP (Control-sh) or GRP75 (GRP75-sh-1 and -2) for 2 d, followed by treatment with 10 µM RA or vehicle alone (0.1% DMSO) for 1 d. Clarified lysates containing equivalent amounts of proteins were analyzed by Western blotting with anti-GRP75 (upper panel) or GAPDH (lower panel, protein load control).
(TIF)

Figure S5 **Overexpression of GRP75 in SH-SY5Y cells decreases the ubiquitination of RARα and RXRα in response to RA signaling.** SH-SY5Y cells were transiently transfected with an GRP75-expressing vector or an empty vector for 48 h, followed by treatment with 10 µM of MG132 in the presence or absence of 10 µM RA for an additional 16 h. Clarified lysates were subjected to immunoprecipitation with a rabbit anti-RARα (A) or anti-RXRα (B) antibody. Proteins pulled down by immunoprecipitation were resolved by SDS-PAGE and analyzed by immunoblotting with an anti-ubiquitin antibody. The same blots were stripped and re-probed with anti-RARα (A, lower panel) or anti-RXRα (B, lower panel) to visualize the individual receptors as loading controls.
(TIF)

Figure S6 **The interaction between GRP75 and RARα/RXRα heterodimers was inversely correlated to tumor volume and tumor weight in a xenograft NB mouse model.** The interaction between GRP75 and RARα/RXRα in xenograft NB tumors harvested from mice treated with RA (solid bar) or saline (open bar) was determined as described in Fig. 6 of the main text. The ratio of RARα to GRP75 (A) or RXRα to GRP75 (B) in individual xenografts was normalized to tumor volume (left panel) or tumor weight (right panel). Quantitative results are shown as the means (±SEM) from xenografts in controls (no treatment, n = 8) or treated animals (RA treatment, n = 8) and were analyzed by Student's t test. *p<0.05.
(TIF)

Figure S7 **A model delineating the GRP75-mediated regulation of RA-elicited neuronal differentiation through direct interaction with RARα/RXRα and the structure modeling predicting interaction interfaces between GRP75 and RARα/RXRα.** (A) The model illustrates that GRP75 could act as a cofactor of RARα/RXRα to mediate RA-triggered neuronal differentiation. Upon RA stimulation, GRP75 could be recruited to the ligand-bound RARα/RXRα heterodimers and cooperatively regulate the expression of RA downstream genes, resulting in enhanced neuronal differentiation. Simultaneously, RA-bound GRP75/RARα/RXRα tripartite complexes could avert UPS-mediated degradation, extending the RA-elicited transactivation of RARα/RXRα to induce neuronal differentiation. (B and C) Structure modeling predicts that GRP75 could bind to the ligand-binding domain (B) or the DNA-binding domain (C) of RARα/RXRα heterodimers. White, GRP75; Green, RARα; Blue, RXRα; Yellow and pink, double strand DNA.
(TIF)

Figure S8 **The binding of GRP75 to RARα or RXRα is elevated in NB patients with favorable outcome.** The levels of GRP75/RARα/RXRα complexes in 30 primary tumors with various histologies was analyzed by immunoprecipitation with a mouse anti-GRP75 antibody, followed by immunoblotting with indicated antibodies. Corresponding quantitative analysis of these blots was shown in the Figure 7 of main text. GNB, ganglioneuroblastoma (n = 13, G1∼G13); DNB, differentiated neuroblastoma (n = 9, D1∼D9); UNB, undifferentiated neuroblastoma (n = 8, U1∼U8).
(TIF)

Figure S9 **Down-regulation of GRP75 abrogates RA-elicited transcriptional activation of RA receptors in SK-N-SH cells.** (A–D) SK-N-SH cells were infected with lentivirus encoding shRNA targeting GFP (Control-sh) or GRP75 (GRP75-sh-1 and -2) for 2 d. The knockdown efficiency was verified by immunoblotting (A) or real-time RT-PCR (inset in F). For promoter assay, the infected cells were additionally transfected with RARE-Luc (B), *RARβ* promoter-Luc (C), NEDD9-Luc (D), or the MYCN-Luc (E) reporter gene construct for an additional 2 d. Following treatment with RA (10 µM) or vehicle alone (0.1% DMSO) for 24 h at 37°C, the luciferase signals in clarified lysates of treated cells were determined and normalized with protein concentration. Normalized luciferase signal of DMSO-treated Control-sh-infected cells were referred to as one fold of relative luciferase activity. (F-G) Infected SK-N-SH cells treated with RA or DMSO as described above were harvested and processed for total RNA isolation by TRIzol reagent. Total RNA transcripts of shRNA-infected SK-N-SH cells treated with RA or DMSO were analyzed by real-time RT-PCR for the expression of RA target genes essential for cell proliferation (F) and neuronal differentiation (G). The normalized level of GRP75 transcript in Control-sh-infected DMSO-treated cells was referred to as 1 fold of relative expression. All quantitative data were calculated as the mean (±SEM) from three independent experiments and analyzed by Student's t test. *p<0.05.
(TIF)

Figure S10 **Overexpression of GRP75 strengthens RA-elicited activation of RA receptors in SK-N-SH cells.** (A) SK-N-SH cells were transiently transfected with an empty vector or a GRP75-expression vector for 24 h. Ectopic expression of GFP-GRP75 in RA-treated transfected cells was analyzed by Western blot analysis with anti-GFP antibody (upper panel, for GRP75). GAPDH (lower panel) was used as a protein loading control. (B and C) SK-N-SH cells were transiently co-transfected with a RA-responsive reporter gene construct (2 mg of RARE-Luc or RARβ-Luc) and a GRP75-expressing construct at various concentrations for 24 h, followed by treatment with 10 µM RA for 24 h. Luciferase signals derived from reporter gene constructs were determined by Steady-Glo luciferase assay reagents and normalized by protein concentration. The normalized luciferase signal in DMSO-treated cells transfected with an empty vector alone was referred to as 1 fold of relative luciferase activity. Quantitative results are presented as the mean (±SEM) of triplicate measurements from three independent experiments and were analyzed by Student's t test. *p<0.05. (D) The levels of GRP75 mRNA transcripts in SK-N-SH cells transiently transfected with a GRP75-expressing vector were determined by quantitative real-time RT-PCR. The normalized level of GRP75 transcripts in DMSO-treated cells transfected with an empty vector alone was referred to as 1 fold of relative expression. Quantitative results are presented as the mean (±SEM) of triplicate measurements from three independent experiments. (E and F) SK-N-SH cells were transfected with an empty vector or a GRP75-expressing vector for 48 h, followed by treatment with or without RA for 24 h. The transcript levels of various RA-responsive genes in transfected cells were determined by quantitative real-time RT-PCR. The normalized transcript level in DMSO-treated cells transfected with an empty vector alone was referred to as 1 fold of relative expression. Quantitative results are presented as the mean (±SEM) of triplicate measurements from three independent experiments and were analyzed by Student's t test.
(TIF)

Figure S11 **The specificity of the mouse anti-GRP75 antibody for co-immunoprecipitation is validated in cultured cells, tumor xenograft, and human primary NB tumors**. The cellular lysates (nuclear and cytosolic fractions, A), homogenates derived from xenografted tumors of mice (B), and clarified extracts derived from human primary NB tumors with different histological grades of differentiation (C) were immunoprecipitated with a mouse anti-GRP75 or a mouse control IgG. The GRP75-bound proteins were analyzed by Western blotting with a goat anti-GRP75, rabbit anti-RARα or rabbit anti-RXRα antibody, respectively. In (C), G, ganglioneuroblastoma; D, differentiated neuroblastoma; U, undifferentiated neuroblastoma.
(TIF)

Figure S12 **RA induces nuclear translocation of GRP75 in SK-N-BE and SK-N-SH cells.** SK-N-DZ (A) and SK-N-SH (B) cells were treated with 10 µM RA for 24 h, the nuclear lysates were subject to Western blot analysis. The levels of nuclear GRP75 were normalized with those of histone H1. The normalized level of GRP75 in cells without RA treatment was referred to as one fold of relative nuclear translocation. All quantitative data were calculated as the mean (±SEM) from three independent experiments.
(TIF)

Figure S13 **RA treatments induce the nuclear translocation of GRP75 in a dose-dependent manner.** SH-SY5Y cells were treated with various concentrations of RA (0.1, 1, and 10 µM) for 24 h, and the nuclear extracts derived from treated cells were analyzed by immunoblotting with an anti-GRP75 or anti-histone H1 (protein loading control of nuclear fraction) antibody. The levels of nuclear GRP75 were normalized with those of histone H1. The normalized level of GRP75 in cells treated with vehicle alone (0.1% DMSO) was referred to as one fold of nuclear GRP75. All quantitative data were calculated as the mean (±SEM) from three independent experiments.
(TIF)

Table S1 The levels of GRP75-bound RARα and RXRα in xenografts in comparison to tumor volume and tumor weight in mice treated with RA or vehicle.
(DOC)

Methods S1 Additional information regarding details of individual experimental procedures.
(DOC)
