# Supplementary figures and images for: Nuclear GRP75 Binds Retinoic Acid Receptors to Promote Neuronal Differentiation of Neuroblastoma
Source: PLoS One. 2011 Oct 14;6(10):e26236. doi: 10.1371/journal.pone.0026236 (PMC3194821; doi:10.1371/journal.pone.0026236)

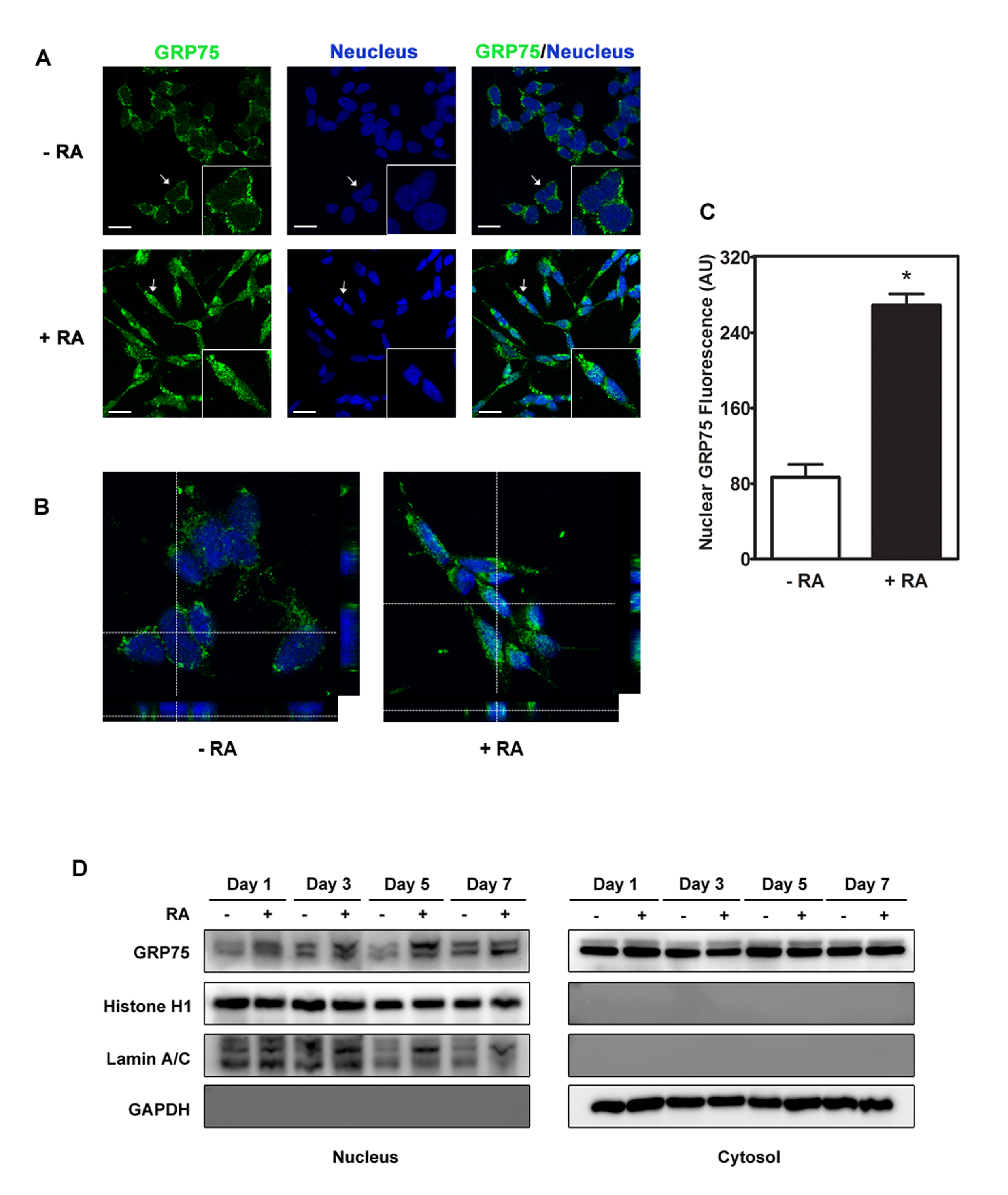

Supplement: Figure S1 — GRP75 is translocated into the nucleus of differentiated neuroblastoma cells. (A) Immunofluorescence microscopy analysis of GRP75 in the nuclei of NB cells. SH-SY5Y cells were treated with or without RA (10 µM) for 3 d and processed for immunofluorescence staining with an anti-GRP75 antibody (green). Nuclei were counterstained with DAPI (blue). Insets, two-fold magnification of highlighted cells (arrow). Scale bar = 20 µm. (B) Three-dimensional analysis of individual cells by z-stack confocal images at specific sites marked by intersecting lines in the x, y, and z axes. (C) Quantitative analysis of the intensity of cells double labeled for GRP75 and DAPI (nuclear DNA). Data are expressed as the average percentage (±SEM) of nuclear GRP75 co-localized with RARa from three independent experiments. *p<0.05. (D) The nuclear extracts of SH-SY5Y cells treated with or without RA for various intervals were resolved by SDS-PAGE and analyzed by immunoblotting with the indicated antibodies. Histone H1 and Lamin A/C were markers for nuclear extracts, while GAPDH was included as a protein loading control for cytosolic pools. The prolonged exposure for GAPDH blot (Nucleus) revealed no contamination of cytosolic proteins in the isolated nuclear extracts. Similarly, overexposure of histone H1- and lamin A/C-labeled blots (Cytosol) showed that isolated cytosolic pools were free from contamination of nuclear proteins. (TIF) [file pone.0026236.s001.tif]

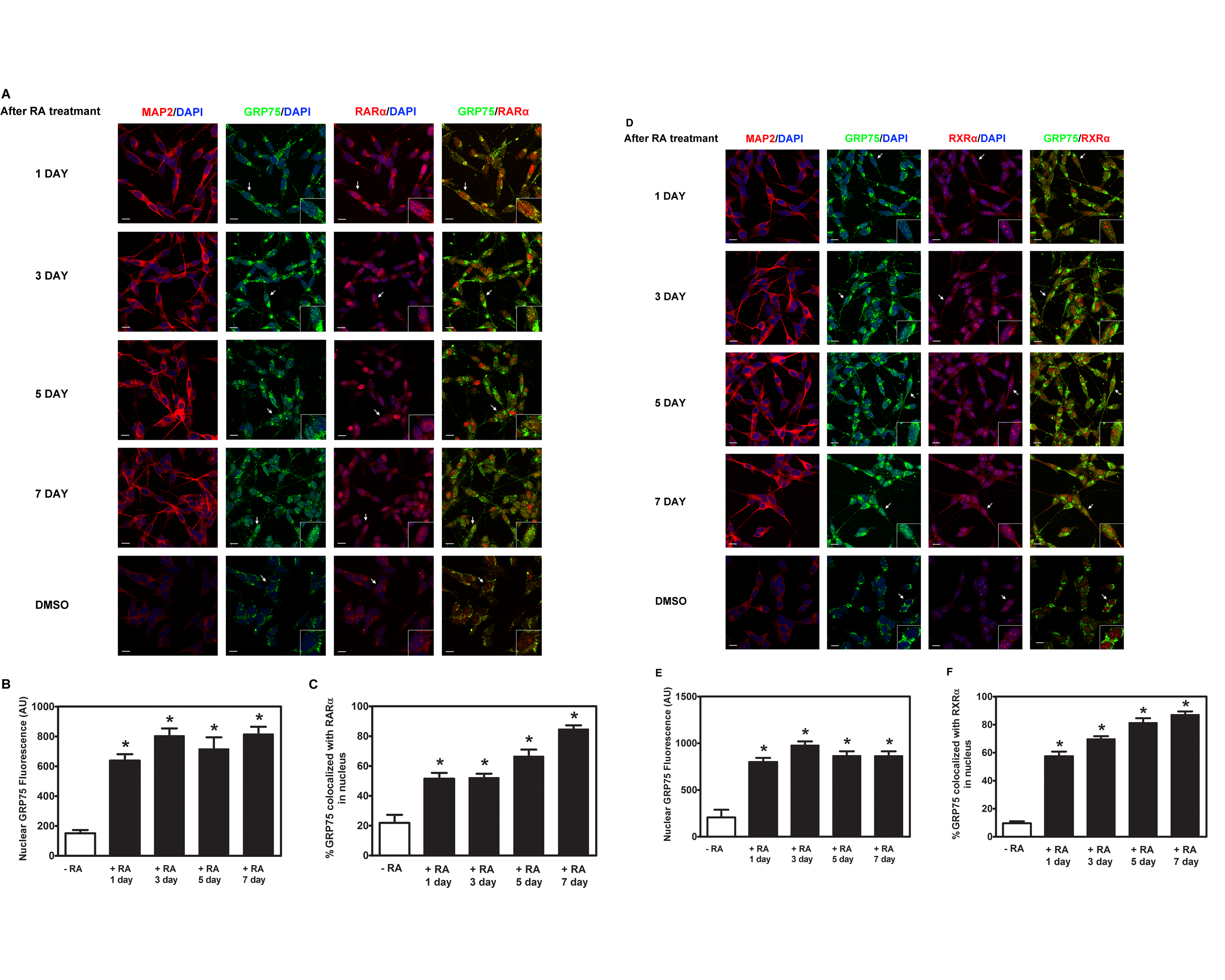

Supplement: Figure S2 — The interaction between GRP75 and RARα/RXRα is increased in RA-treated SH-SY5Y cells. (A) SH-SY5Y cells were grown on coverslips and treated with 10 µM RA for various intervals. Cells treated with vehicle alone (0.1% DMSO) were included as controls. Treated cells were fixed with 4% paraformaldehyde and subjected to immunofluorescence staining using goat anti-GRP75 (green), mouse anti-MAP2 (red), and rabbit anti-RARα (red). Nuclei were counterstained with DAPI. The inset shows the magnification of the highlighted region (arrow). Scale bar = 20 µm. (B) The levels of nuclear GRP75 were quantified using MetaMorph Offline 7.5.1.0 Image Analysis System (Molecular Devices). Quantitative results are shown as the mean (±SEM) from three independent experiments. (C) The levels of GRP75 co-localized with RARα in the nucleus were quantified using the MetaMorph Offline 7.5.1.0 Image Analysis System (Molecular Devices). Quantitative data are shown as means (±SEM) of at least three different viewing areas from three independent experiments. All quantitative data were analyzed by Student's t test. *p<0.05 versus DMSO-treated control (– RA). (D) SH-SY5Y cells were treated with 10 µM RA for various intervals. Cells treated with vehicle alone (0.1% DMSO) were included as controls (– RA). Treated cells were fixed with 4% paraformaldehyde and subjected to immunofluorescence staining using goat anti-GRP75 (green), mouse anti-MAP2 (red), and rabbit anti-RXRα (red). Nuclei were visualized by DAPI staining. The inset shows the magnified view of the highlighted region (arrow). Scale bar = 20 µm. (E and F) The levels of nuclear GRP75 (E) and RXRα-co-localized GRP75 in the nucleus (F) were determined using the MetaMorph Offline 7.5.1.0 Image Analysis System (Molecular Devices). Quantitative results are shown as means (±SEM) from three independent experiments and were analyzed by Student's t test. *p<0.05 versus DMSO-treated control (– RA). The number of cells used in the quantitative analysis w [file pone.0026236.s002.tif]

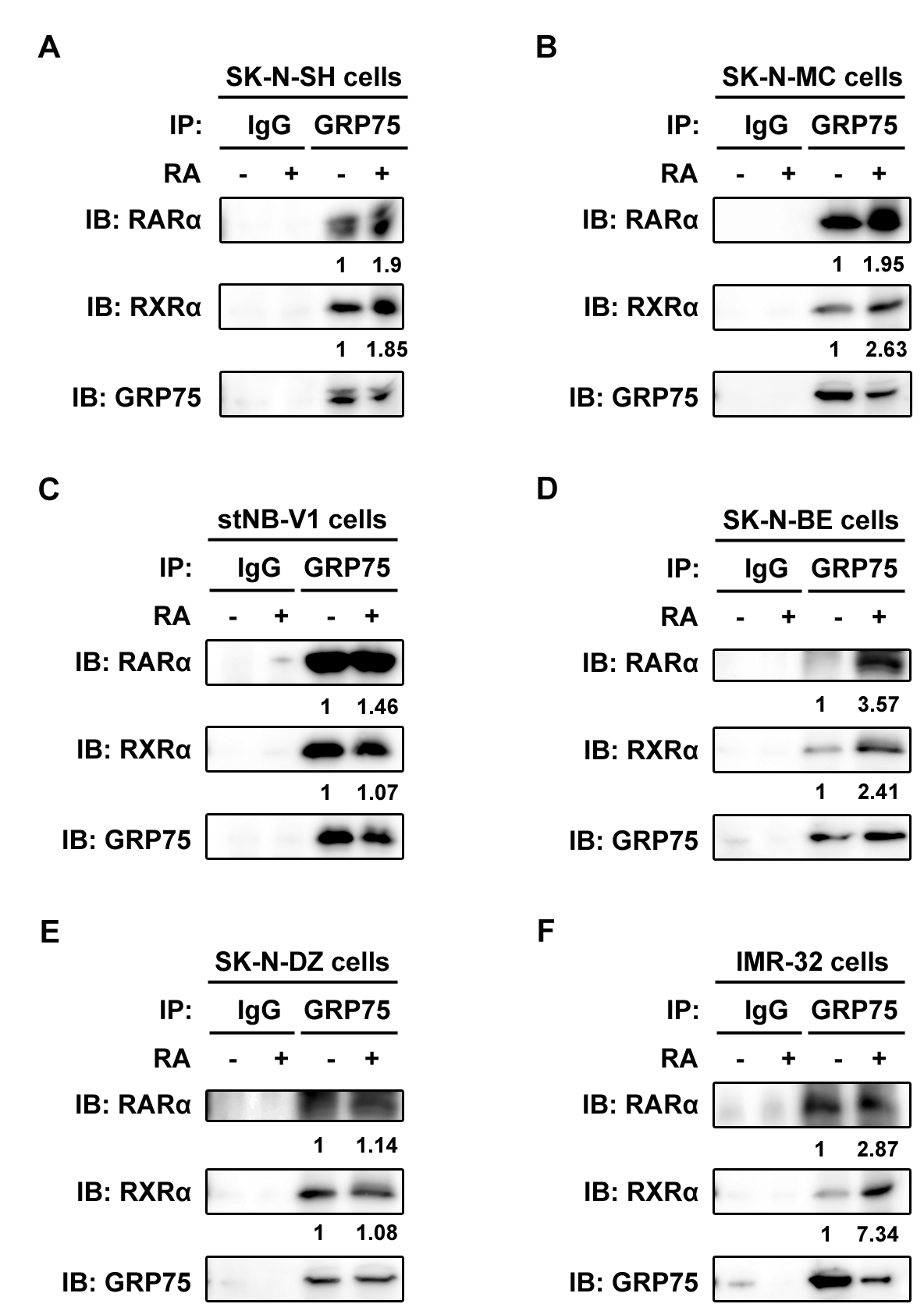

Supplement: Figure S3 — RA enhances the formation of GRP75/RARα/RXRα tripartite complexes in various NB cell lines. MYCN-nonamplified NB cell lines, including SK-N-SH (A), SK-N-MC (B), and stNB-V1 (C), and MYCN-amplified NB cell lines, including SK-N-BE (D), SK-N-DZ (E), and IMR-32 (F), were treated with 10 µM RA or vehicle alone (0.1% DMSO) for 1 d. Nuclear lysates were immunoprecipitated with a mouse anti-GRP75 antibody. Protein A-bound antigen-antibody complexes were analyzed by immunoblotting with anti-RARα (upper panel), anti-RXRα (middle panel), or anti-GRP75 (lower panel, loading control). The levels of RARα and RXRα were normalized to GRP75 from the same immunoprecipitate, and those in cells without RA treatment were referred to as one fold of relative interaction between GRP75 and respective RA receptors (RARα and RXRα). (TIF) [file pone.0026236.s003.tif]

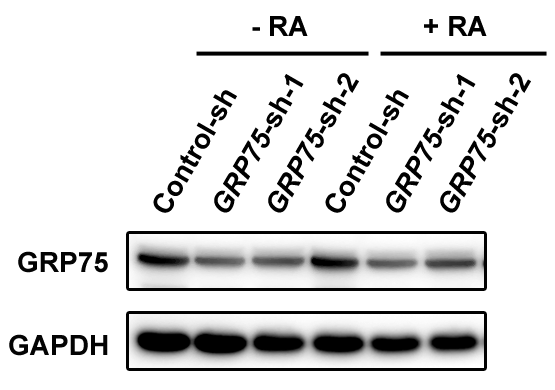

Supplement: Figure S4 — Lentiviral shRNA-mediated down-regulation of GRP75 in SH-SY5Y cells. SH-SY5Y cells were infected with lentiviral shRNA targeting GFP (Control-sh) or GRP75 (GRP75-sh-1 and -2) for 2 d, followed by treatment with 10 µM RA or vehicle alone (0.1% DMSO) for 1 d. Clarified lysates containing equivalent amounts of proteins were analyzed by Western blotting with anti-GRP75 (upper panel) or GAPDH (lower panel, protein load control). (TIF) [file pone.0026236.s004.tif]

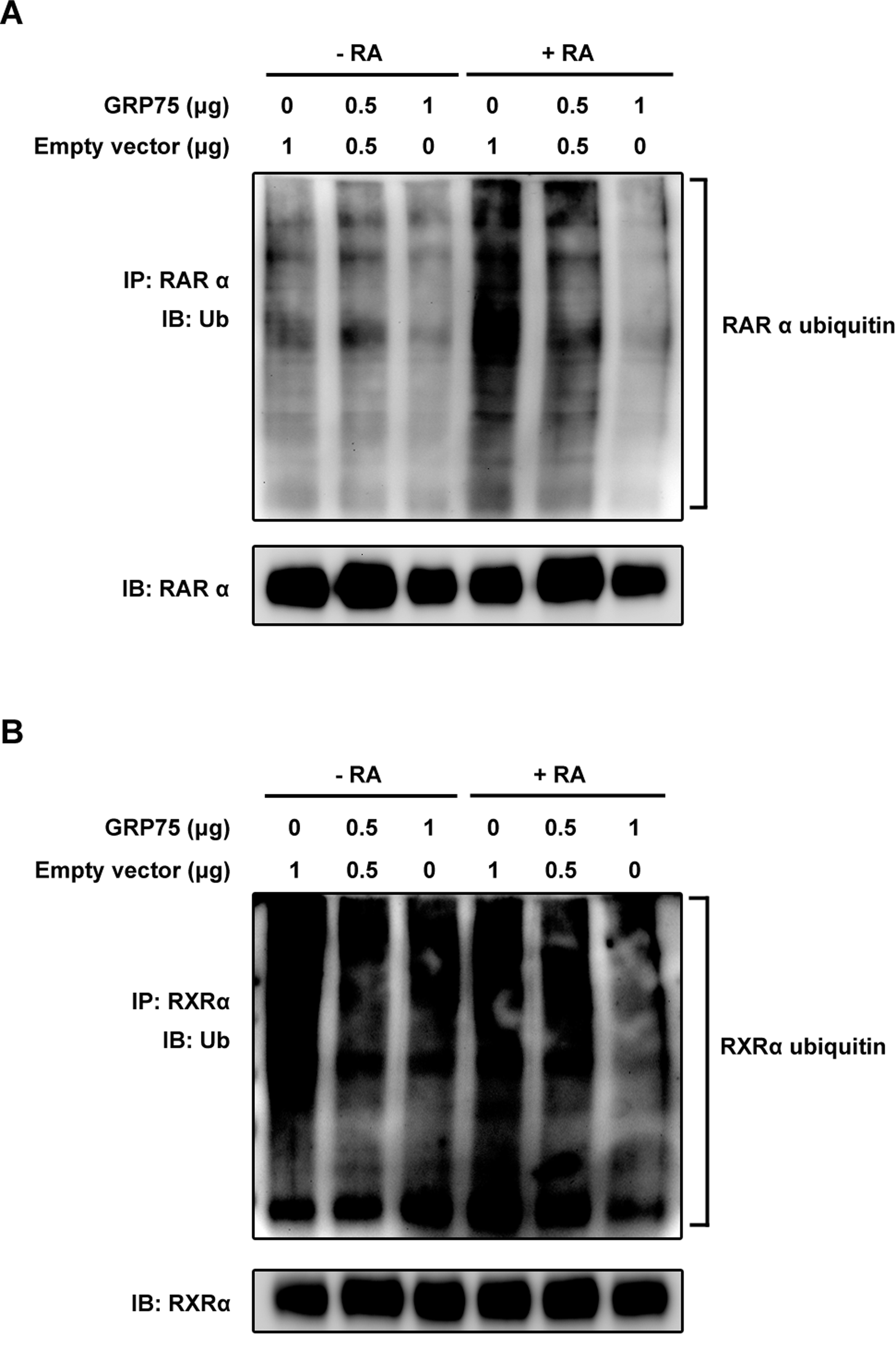

Supplement: Figure S5 — Overexpression of GRP75 in SH-SY5Y cells decreases the ubiquitination of RARα and RXRα in response to RA signaling. SH-SY5Y cells were transiently transfected with an GRP75-expressing vector or an empty vector for 48 h, followed by treatment with 10 µM of MG132 in the presence or absence of 10 µM RA for an additional 16 h. Clarified lysates were subjected to immunoprecipitation with a rabbit anti-RARα (A) or anti-RXRα (B) antibody. Proteins pulled down by immunoprecipitation were resolved by SDS-PAGE and analyzed by immunoblotting with an anti-ubiquitin antibody. The same blots were stripped and re-probed with anti-RARα (A, lower panel) or anti-RXRα (B, lower panel) to visualize the individual receptors as loading controls. (TIF) [file pone.0026236.s005.tif]

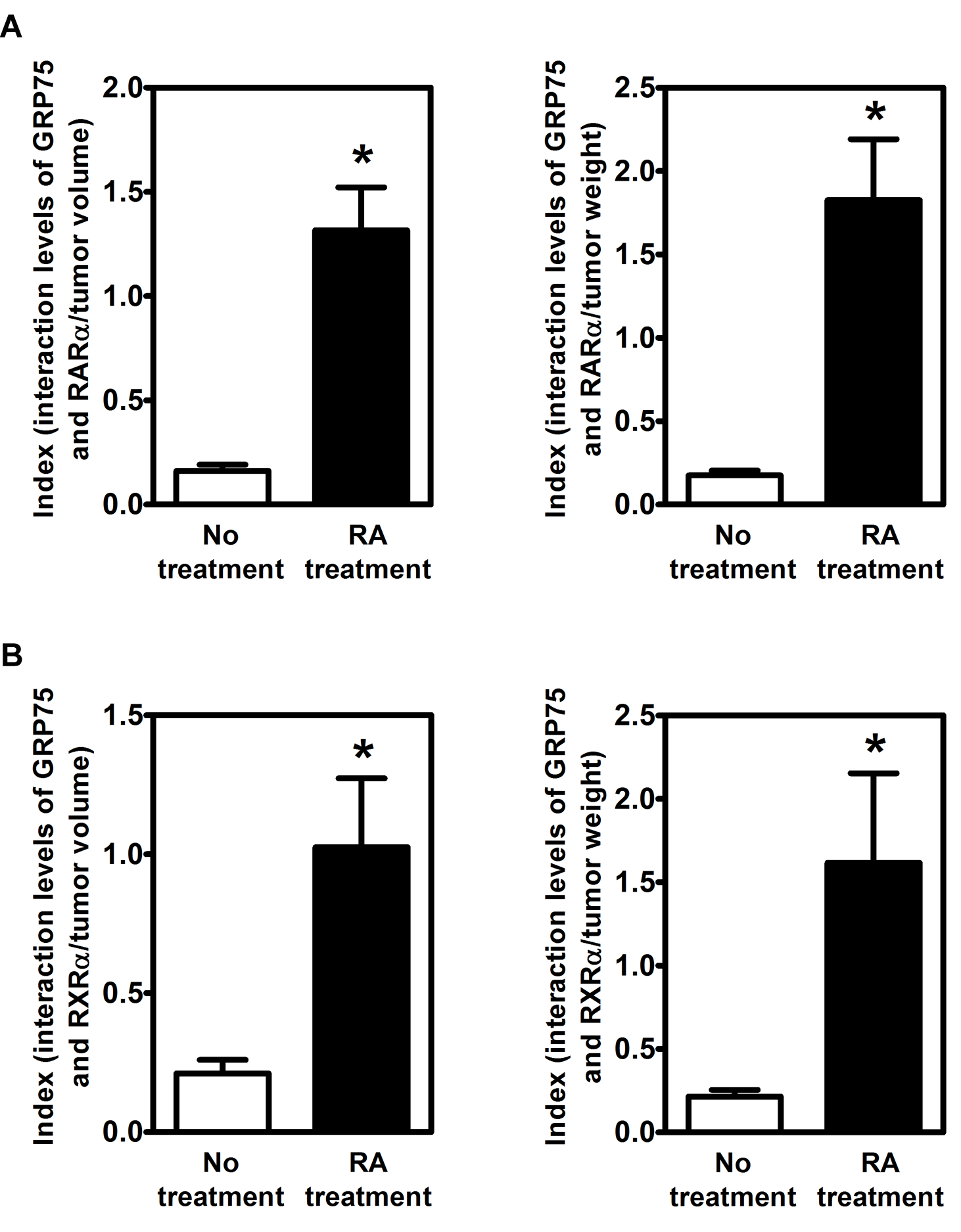

Supplement: Figure S6 — The interaction between GRP75 and RARα/RXRα heterodimers was inversely correlated to tumor volume and tumor weight in a xenograft NB mouse model. The interaction between GRP75 and RARα/RXRα in xenograft NB tumors harvested from mice treated with RA (solid bar) or saline (open bar) was determined as described in Fig. 6 of the main text. The ratio of RARα to GRP75 (A) or RXRα to GRP75 (B) in individual xenografts was normalized to tumor volume (left panel) or tumor weight (right panel). Quantitative results are shown as the means (±SEM) from xenografts in controls (no treatment, n = 8) or treated animals (RA treatment, n = 8) and were analyzed by Student's t test. *p<0.05. (TIF) [file pone.0026236.s006.tif]

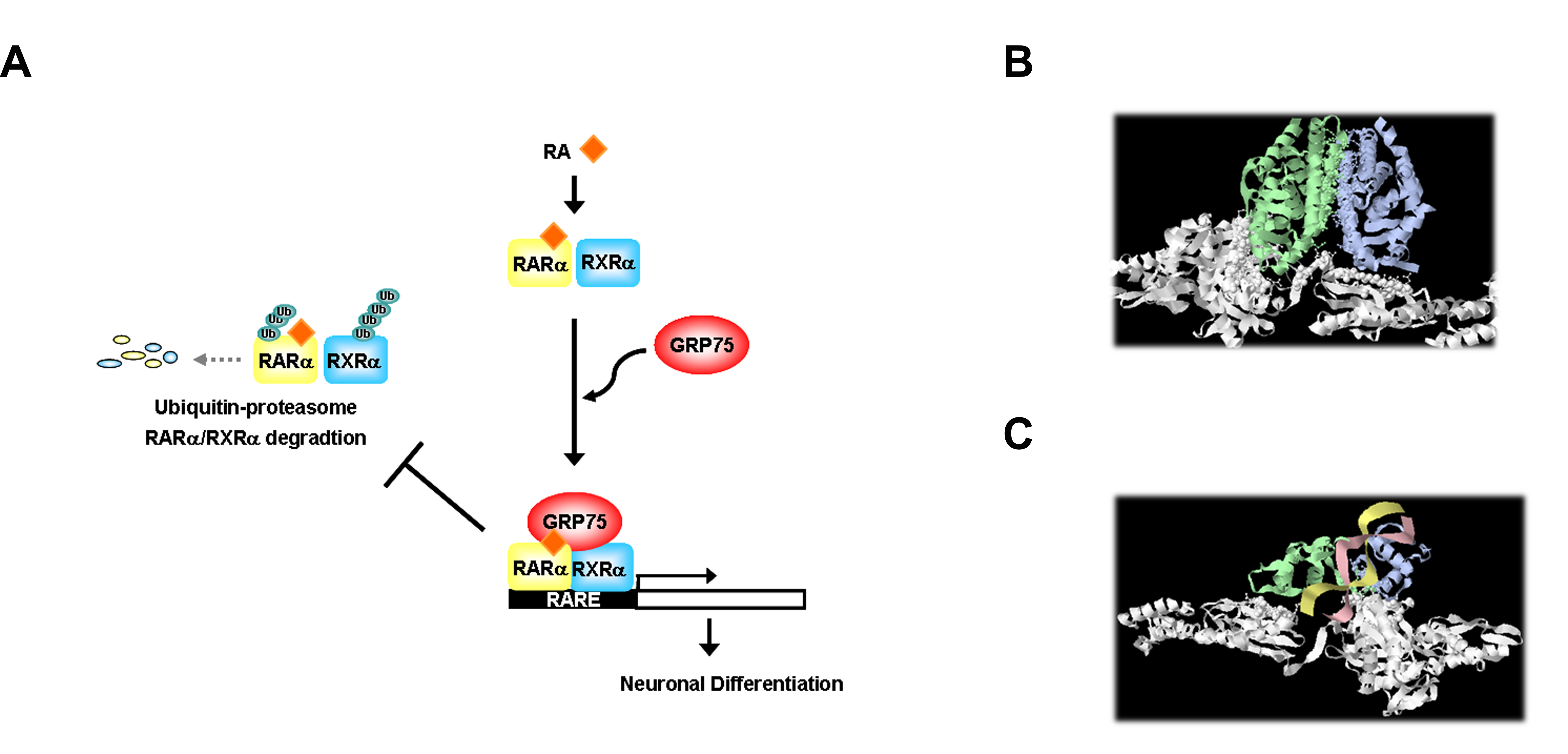

Supplement: Figure S7 — A model delineating the GRP75-mediated regulation of RA-elicited neuronal differentiation through direct interaction with RARα/RXRα and the structure modeling predicting interaction interfaces between GRP75 and RARα/RXRα. (A) The model illustrates that GRP75 could act as a cofactor of RARα/RXRα to mediate RA-triggered neuronal differentiation. Upon RA stimulation, GRP75 could be recruited to the ligand-bound RARα/RXRα heterodimers and cooperatively regulate the expression of RA downstream genes, resulting in enhanced neuronal differentiation. Simultaneously, RA-bound GRP75/RARα/RXRα tripartite complexes could avert UPS-mediated degradation, extending the RA-elicited transactivation of RARα/RXRα to induce neuronal differentiation. (B and C) Structure modeling predicts that GRP75 could bind to the ligand-binding domain (B) or the DNA-binding domain (C) of RARα/RXRα heterodimers. White, GRP75; Green, RARα; Blue, RXRα; Yellow and pink, double strand DNA. (TIF) [file pone.0026236.s007.tif]

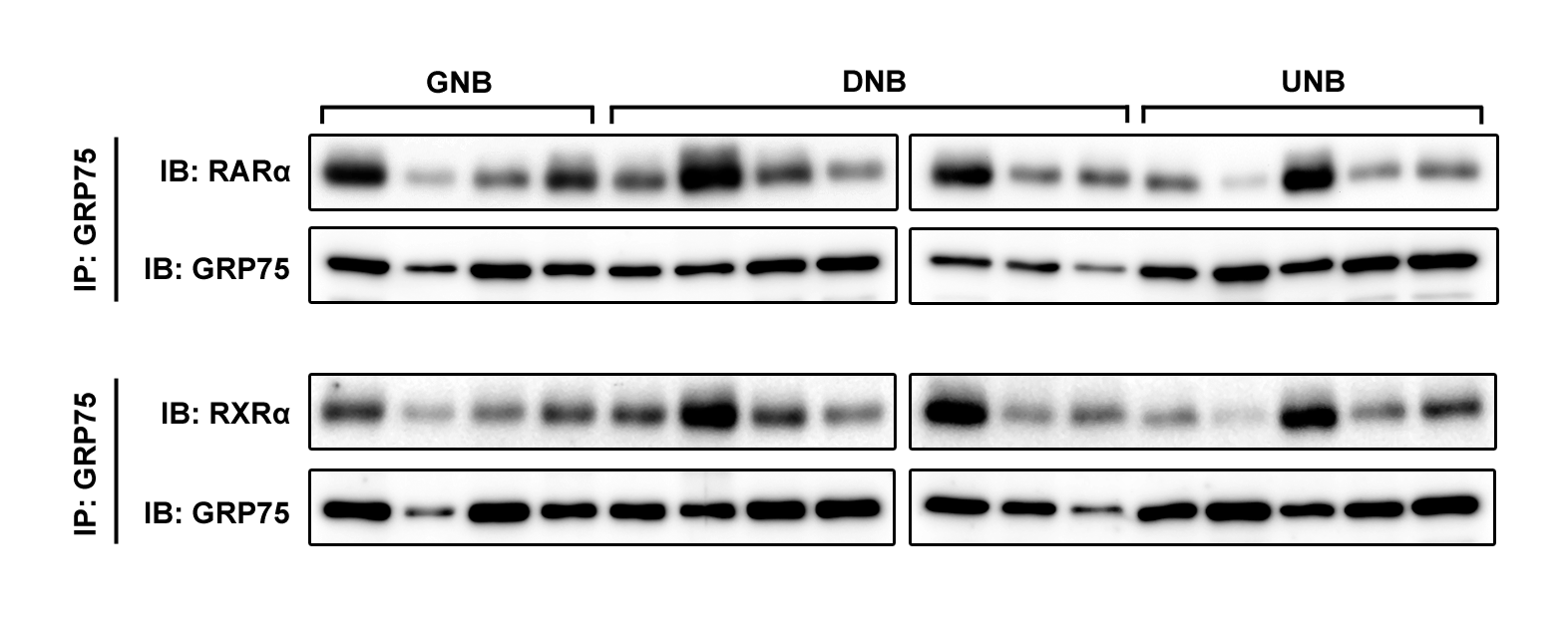

Supplement: Figure S8 — The binding of GRP75 to RARα or RXRα is elevated in NB patients with favorable outcome. The levels of GRP75/RARα/RXRα complexes in 30 primary tumors with various histologies was analyzed by immunoprecipitation with a mouse anti-GRP75 antibody, followed by immunoblotting with indicated antibodies. Corresponding quantitative analysis of these blots was shown in the Figure 7 of main text. GNB, ganglioneuroblastoma (n = 13, G1∼G13); DNB, differentiated neuroblastoma (n = 9, D1∼D9); UNB, undifferentiated neuroblastoma (n = 8, U1∼U8). (TIF) [file pone.0026236.s008.tif]

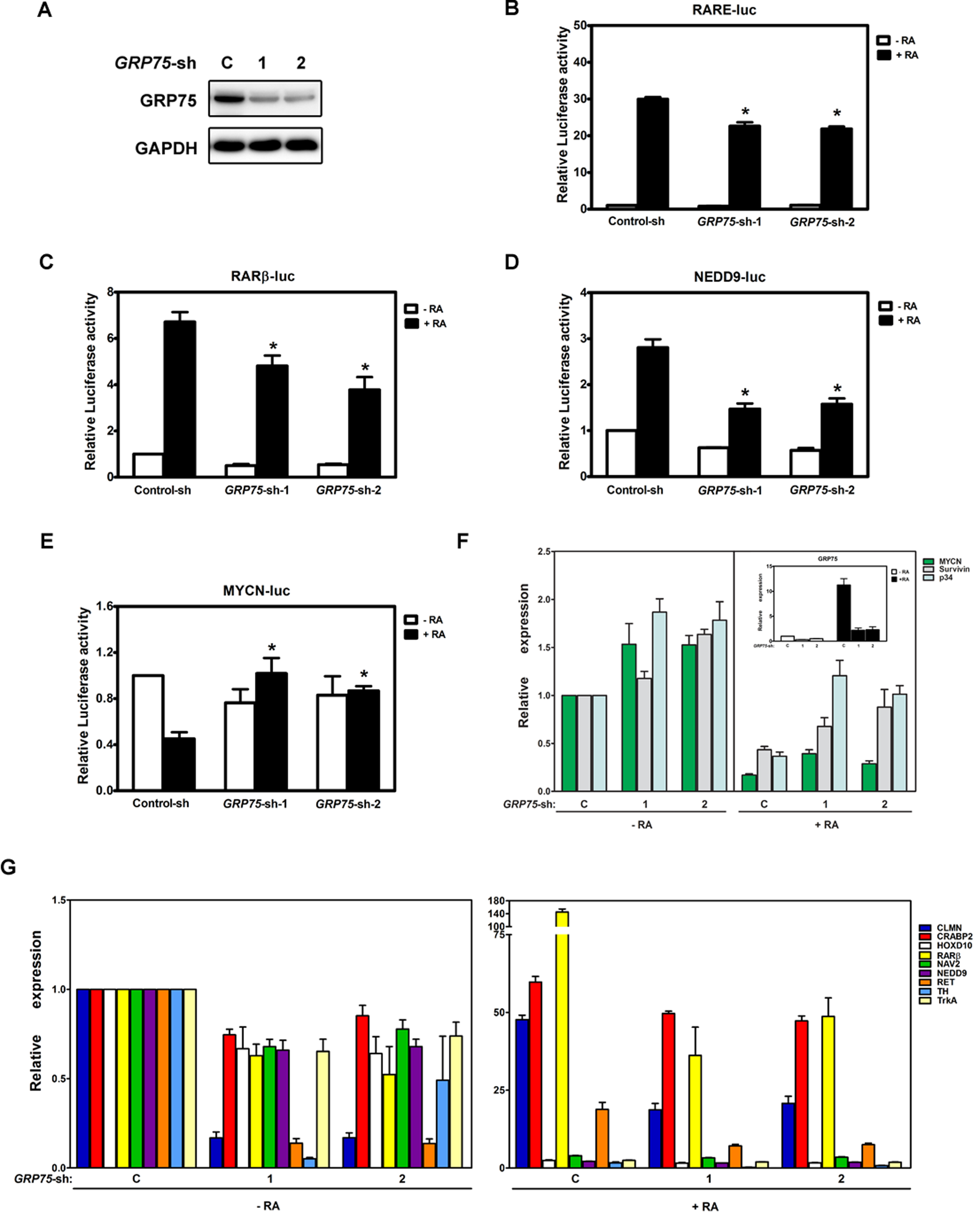

Supplement: Figure S9 — Down-regulation of GRP75 abrogates RA-elicited transcriptional activation of RA receptors in SK-N-SH cells. (A–D) SK-N-SH cells were infected with lentivirus encoding shRNA targeting GFP (Control-sh) or GRP75 (GRP75-sh-1 and -2) for 2 d. The knockdown efficiency was verified by immunoblotting (A) or real-time RT-PCR (inset in F). For promoter assay, the infected cells were additionally transfected with RARE-Luc (B), RARβ promoter-Luc (C), NEDD9-Luc (D), or the MYCN-Luc (E) reporter gene construct for an additional 2 d. Following treatment with RA (10 µM) or vehicle alone (0.1% DMSO) for 24 h at 37°C, the luciferase signals in clarified lysates of treated cells were determined and normalized with protein concentration. Normalized luciferase signal of DMSO-treated Control-sh-infected cells were referred to as one fold of relative luciferase activity. (F-G) Infected SK-N-SH cells treated with RA or DMSO as described above were harvested and processed for total RNA isolation by TRIzol reagent. Total RNA transcripts of shRNA-infected SK-N-SH cells treated with RA or DMSO were analyzed by real-time RT-PCR for the expression of RA target genes essential for cell proliferation (F) and neuronal differentiation (G). The normalized level of GRP75 transcript in Control-sh-infected DMSO-treated cells was referred to as 1 fold of relative expression. All quantitative data were calculated as the mean (±SEM) from three independent experiments and analyzed by Student's t test. *p<0.05. (TIF) [file pone.0026236.s009.tif]

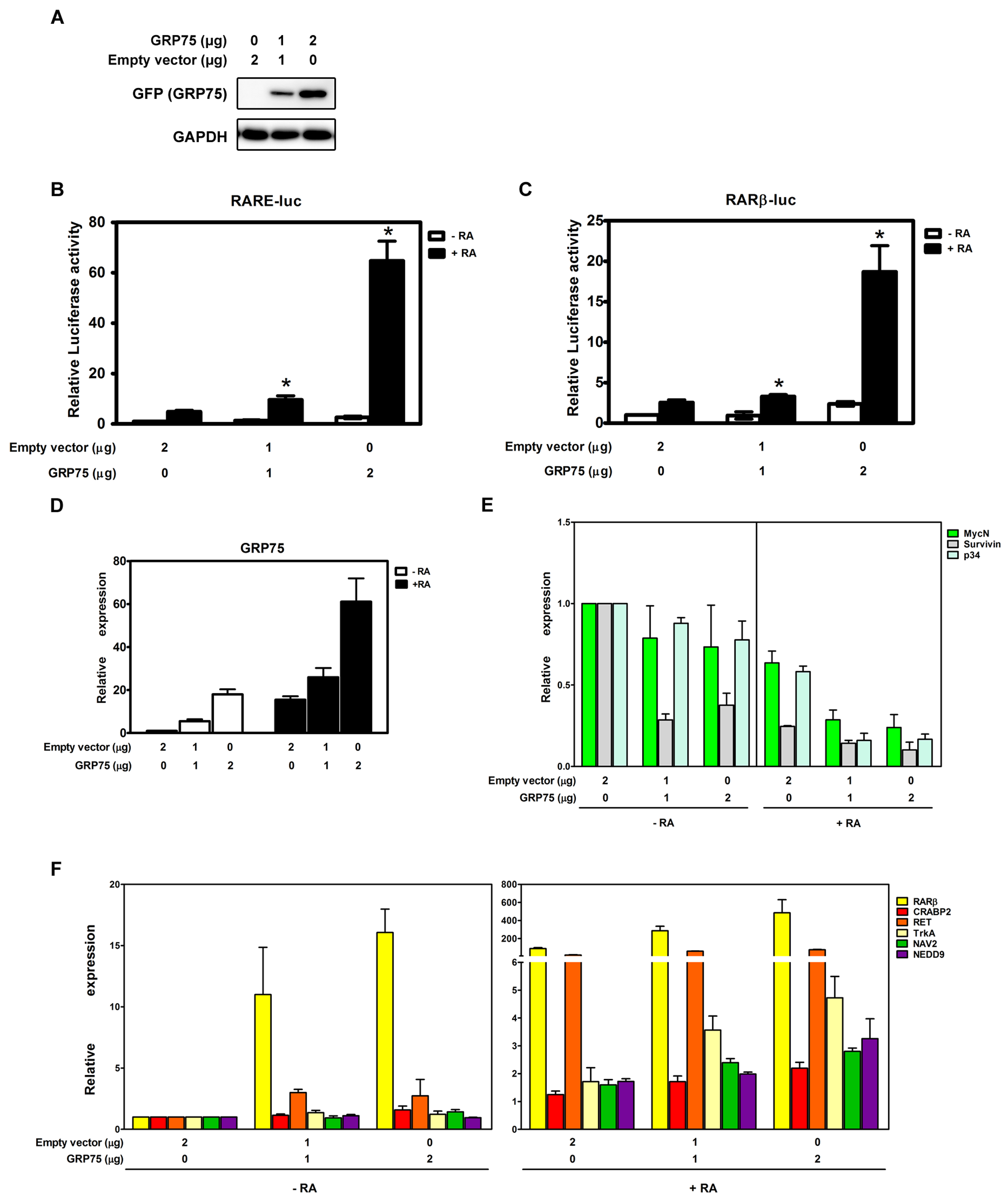

Supplement: Figure S10 — Overexpression of GRP75 strengthens RA-elicited activation of RA receptors in SK-N-SH cells. (A) SK-N-SH cells were transiently transfected with an empty vector or a GRP75-expression vector for 24 h. Ectopic expression of GFP-GRP75 in RA-treated transfected cells was analyzed by Western blot analysis with anti-GFP antibody (upper panel, for GRP75). GAPDH (lower panel) was used as a protein loading control. (B and C) SK-N-SH cells were transiently co-transfected with a RA-responsive reporter gene construct (2 mg of RARE-Luc or RARβ-Luc) and a GRP75-expressing construct at various concentrations for 24 h, followed by treatment with 10 µM RA for 24 h. Luciferase signals derived from reporter gene constructs were determined by Steady-Glo luciferase assay reagents and normalized by protein concentration. The normalized luciferase signal in DMSO-treated cells transfected with an empty vector alone was referred to as 1 fold of relative luciferase activity. Quantitative results are presented as the mean (±SEM) of triplicate measurements from three independent experiments and were analyzed by Student's t test. *p<0.05. (D) The levels of GRP75 mRNA transcripts in SK-N-SH cells transiently transfected with a GRP75-expressing vector were determined by quantitative real-time RT-PCR. The normalized level of GRP75 transcripts in DMSO-treated cells transfected with an empty vector alone was referred to as 1 fold of relative expression. Quantitative results are presented as the mean (±SEM) of triplicate measurements from three independent experiments. (E and F) SK-N-SH cells were transfected with an empty vector or a GRP75-expressing vector for 48 h, followed by treatment with or without RA for 24 h. The transcript levels of various RA-responsive genes in transfected cells were determined by quantitative real-time RT-PCR. The normalized transcript level in DMSO-treated cells transfected with an empty vector alone was referred to as 1 fold of relative expression. Quantitative results [file pone.0026236.s010.tif]

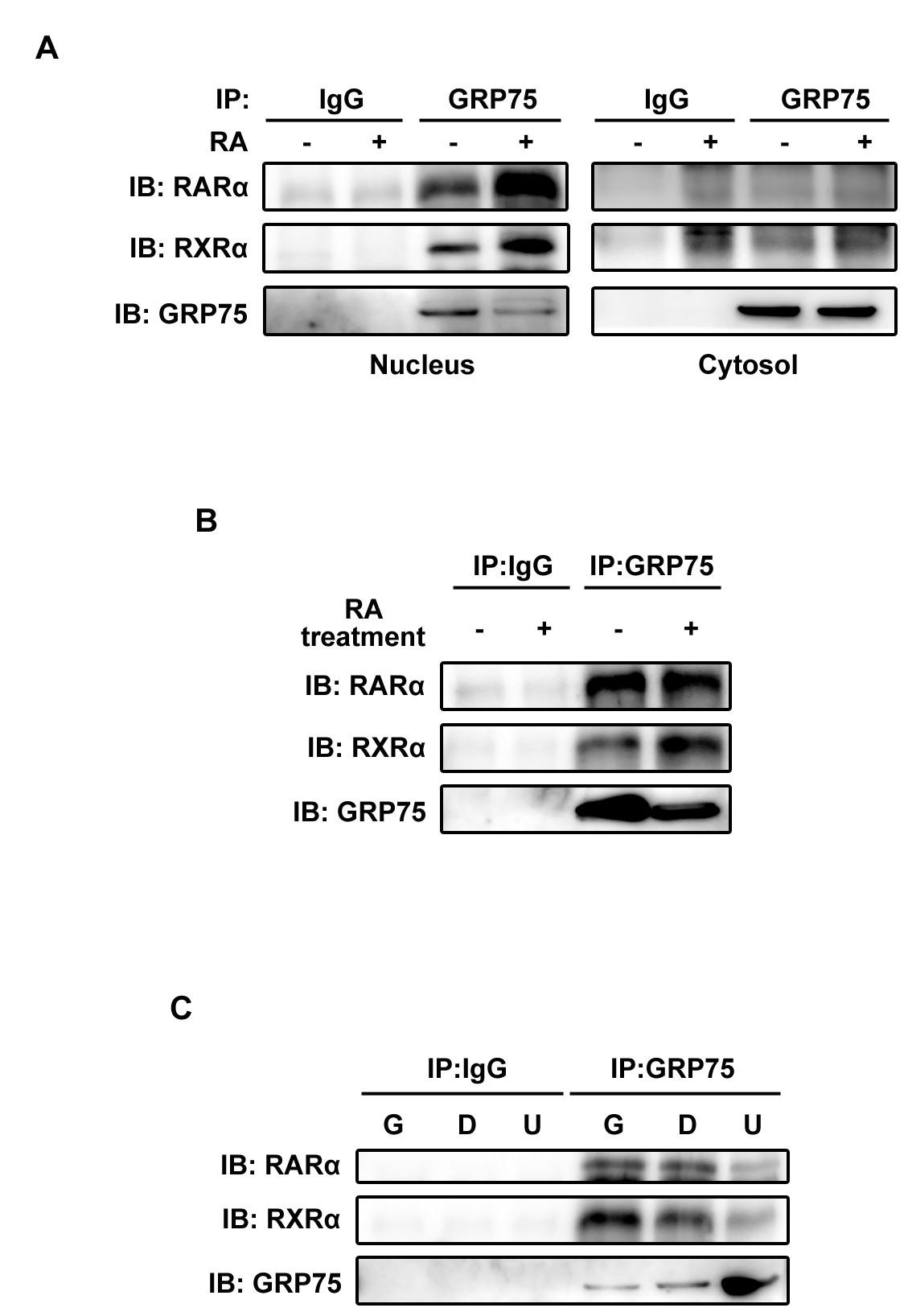

Supplement: Figure S11 — The specificity of the mouse anti-GRP75 antibody for co-immunoprecipitation is validated in cultured cells, tumor xenograft, and human primary NB tumors. The cellular lysates (nuclear and cytosolic fractions, A), homogenates derived from xenografted tumors of mice (B), and clarified extracts derived from human primary NB tumors with different histological grades of differentiation (C) were immunoprecipitated with a mouse anti-GRP75 or a mouse control IgG. The GRP75-bound proteins were analyzed by Western blotting with a goat anti-GRP75, rabbit anti-RARα or rabbit anti-RXRα antibody, respectively. In (C), G, ganglioneuroblastoma; D, differentiated neuroblastoma; U, undifferentiated neuroblastoma. (TIF) [file pone.0026236.s011.tif]

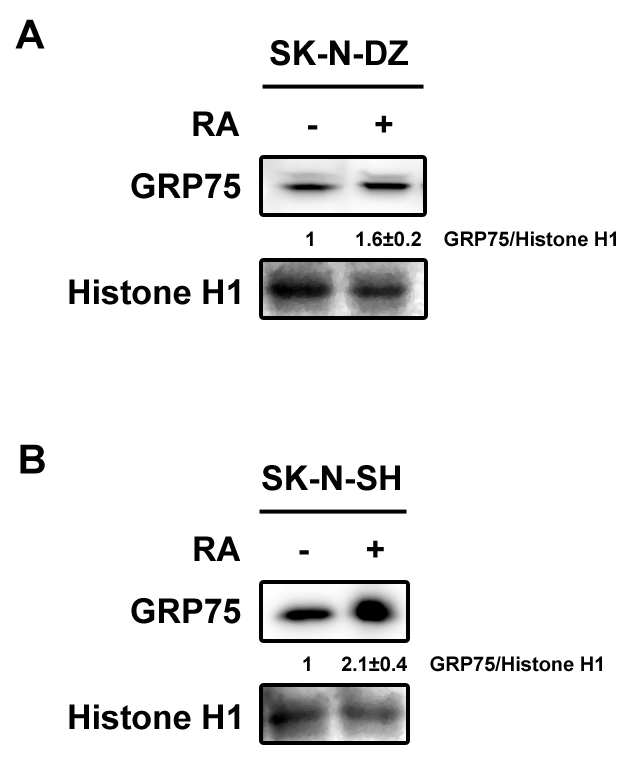

Supplement: Figure S12 — RA induces nuclear translocation of GRP75 in SK-N-BE and SK-N-SH cells. SK-N-DZ (A) and SK-N-SH (B) cells were treated with 10 µM RA for 24 h, the nuclear lysates were subject to Western blot analysis. The levels of nuclear GRP75 were normalized with those of histone H1. The normalized level of GRP75 in cells without RA treatment was referred to as one fold of relative nuclear translocation. All quantitative data were calculated as the mean (±SEM) from three independent experiments. (TIF) [file pone.0026236.s012.tif]

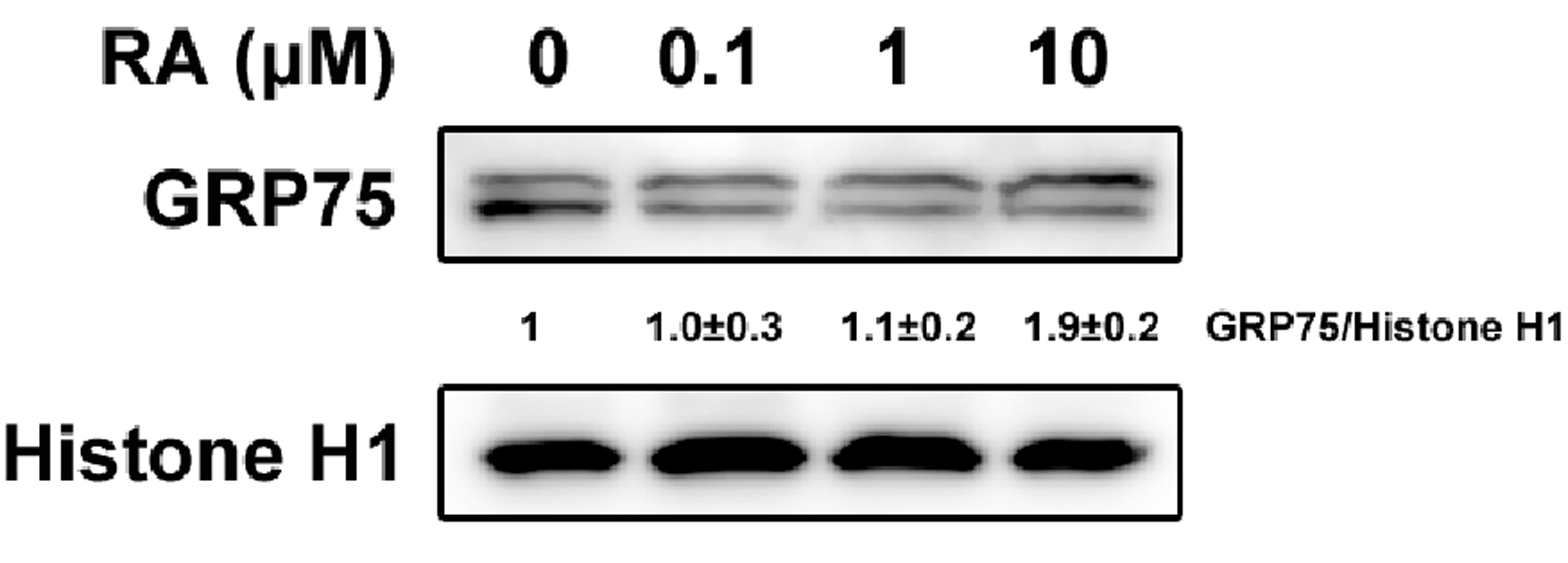

Supplement: Figure S13 — RA treatments induce the nuclear translocation of GRP75 in a dose-dependent manner. SH-SY5Y cells were treated with various concentrations of RA (0.1, 1, and 10 µM) for 24 h, and the nuclear extracts derived from treated cells were analyzed by immunoblotting with an anti-GRP75 or anti-histone H1 (protein loading control of nuclear fraction) antibody. The levels of nuclear GRP75 were normalized with those of histone H1. The normalized level of GRP75 in cells treated with vehicle alone (0.1% DMSO) was referred to as one fold of nuclear GRP75. All quantitative data were calculated as the mean (±SEM) from three independent experiments. (TIF) [file pone.0026236.s013.tif]
