# Supplementary material for: Nuclear GRP75 Binds Retinoic Acid Receptors to Promote Neuronal Differentiation of Neuroblastoma
Source: PLoS One. 2011 Oct 14;6(10):e26236. doi: 10.1371/journal.pone.0026236 (PMC3194821; doi:10.1371/journal.pone.0026236)
Supplement: Table S1 — The levels of GRP75-bound RARα and RXRα in xenografts in comparison to tumor volume and tumor weight in mice treated with RA or vehicle. (DOC) [file pone.0026236.s014.doc]

**Table S1. The levels of GRP75-bound RARα and RXRα in xenografts in comparison to tumor volume and tumor weight in mice treated with RA or vehicle.**

| **Group GRP75/RARα GRP75/RXRα Tumor volume (cm^3^) Tumor weight (g)** |
| --- |
| **Vehicle** 0.439 ± 0.10 0.543 ± 0.12 2.670 ± 0.41 2.476 ± 0.30  **RA** 1.819 ± 0.22* 1.405 ± 0.27* 1.494 ± 0.13* 1.105 ± 0.14* |
| Results are expressed as the means ± SEM. Vehicle, n = 8; RA, n = 8.  * P < 0.0 versus vehicle. |
