# Supplementary material for: Nuclear GRP75 Binds Retinoic Acid Receptors to Promote Neuronal Differentiation of Neuroblastoma
Source: PLoS One. 2011 Oct 14;6(10):e26236. doi: 10.1371/journal.pone.0026236 (PMC3194821; doi:10.1371/journal.pone.0026236)
Supplement: Methods S1 — Additional information regarding details of individual experimental procedures. (DOC) [file pone.0026236.s015.doc]

***Supplemental Methods***

**Nuclear GRP75 Binds Retinoic Acid Receptors to Promotes Neuronal Differentiation of Neuroblastoma**

Yu-Yin Shih^1,2^, Hsinyu Lee^2,3^, Akira Nakagawara^5^, Hseuh-Fen Juan^3,4^, Yung-Ming Jeng^6^, Yeou-Guang Tsay^7^, Dong-Tsamn Lin^8,11^, Fon-Jou Hsieh^9^, Chien-Yuan Pan^2,3^*, Wen-Ming Hsu^10,11^*, and Yung-Feng Liao^1^*

^1^Laboratory of Molecular Neurobiology, Institute of Cellular and Organismic Biology, Academia Sinica, Taipei 11529, Taiwan; ^2^Institutes of Zoology, ^3^Department of Life Science, and ^4^Institute of Molecular and Cellular Biology, National Taiwan University, Taipei 10617, Taiwan; ^5^Chiba Cancer Center Research Institute, Department of Molecular Biology and Oncology, Chiba University Graduate School of Medicine, Chiba 260-8717, Japan; ^7^Institute of Biochemistry and Molecular Biology and Proteomics Research Center, National Yang-Ming University, Taipei 11221, Taiwan; ^9^Graduate Institute of Clinical Medicine, National Taiwan University College of Medicine, Taipei 10051, Taiwan; Departments of ^6^Pathology, ^8^Pediatrics, and ^10^Surgery, National Taiwan University Hospital and National Taiwan University College of Medicine, Taipei 10002, Taiwan; ^11^Childhood Cancer Foundation, Taipei 10041, Taiwan.

**Supplemental Experimental Procedures**

**Cell culture**

The human neuroblastoma cell line (SH-SY5Y) was purchased from American Type Culture Collection (ATCC). SH-SY5Y cells were maintained in DMEM/F12 medium supplemented with 10% FBS. For the generation of a xenograft mouse model, a GFP-expressing NB cell line stNA-V1 was cultured in DMEM/F-12 supplemented with 10% FBS and 400 mg/ml G418 [1]. Cells were incubated in a humidified incubator at 37 ºC in 5% CO_2_.

**Reagents and antibodies**

All-trans retinoic acid (RA) was from Sigma. MG132 and cycloheximide were purchased from Calbiochem. Immobilized protein A agarose was obtained from Pierce. Antibodies against GRP75 (C-19), RARα (C-20), RXRα (D-20), histone H1 (AE-4), GAPDH (FL-335), lamin A/C (636), and rabbit IgG were from Santa Cruz Biotechnology. An anti-MAP2 (AP20) antibody was purchased from Millipore, and the anti-ubiquitin (P4D1) antibody was from Cell signaling. A mouse anti-GRP75 antibody was from Affinity BioReagents. All other reagents were at least reagent grade and obtained from standard suppliers.

**Immunofluorescence confocal microscopy**

SH-SY5Y cells were grown on coverslips and treated with 10 μM RA in DMSO or 0.1% DMSO alone for the indicated time. Immunofluorescence staining was performed as described previously [2]. Briefly, cells were fixed with 4% paraformaldelhyde for 1 h and permeabilized with phosphate-buffered saline (PBS) containing 0.1% Triton-X100 for 10 min. The cells were washed in PBS three times and blocked in 2% BSA for 1 h, followed by incubation with a goat anti-GRP75 antibody (1:200) overnight at 4 °C. Following the removal of unbound antibodies, cells were incubated with an Alexa 488-conjugated donkey anti-goat IgG (1:200) for 1 h. For the double immunostaining of GRP75 and either RARα or RXRα, cells were further incubated with either rabbit anti-RARα or anti-RXRα antibody (1:200) for 1 h at room temperature, followed by incubation with an Alexa 555-conjugated goat anti-rabbit IgG (1:200) for 1 h at room temperature. For the visualization of the neuronal differentiation marker MAP2, cells were further incubated with a mouse anti-MAP2 antibody (1:300) for 1 h and then an Alexa 647-labeled goat anti-mouse IgG antibody (1:200) for 1 h. The nuclei were visualized by counterstaining with 4,6-diamidino-2-phenylindole (DAPI) for 5 min. Following extensive washing with PBS, the slides were mounted with Vectashield (Vector Laboratories). The fluorescence images were acquired with a laser scanning confocal microscope system (Leica TCS-AOBS-SP5) with a 63X oil-immersion objective. The fluorescence intensity of GRP75 was measured using Leica Application Suite Advanced Fluorescence. Merged images were generated by Adobe Photoshop CS3, and the nuclear co-localization of two selected proteins was analyzed with the MetaMorph Offline 7.5.1.0 Image Analysis System (Molecular Devices). Quantitative data were analyzed by Student’s t test.

**Generation of the GFP-tagged GRP75 expression vector, plasmid transfection, and lentiviral infection**

The full length GRP75 cDNA was amplified from human brain poly-A^+^ mRNA (Clontech) by PCR with forward and reverse primers (5´-CCC TCG AGC TAT GAT AAG TGC CAG CCG AGC TGC AGC AGC C-3´ and 5´-CCG GAT CCC TGT TTT TCC TCC TTT TGA TCT TCC TT-3´) and subcloned into the *Xho* I/*Bam* HI site of the pEGFP-C1 plasmid (Clontech) to generate GFP-GRP75 constructs. The DR5-TK and RARβ-luciferase reporter constructs were kindly provided by Dr. Jonathan Kurie (The University of Texas M.D. Anderson Cancer Center) [3]. The Nedd9 promoter constructs were kindly provided by Dr. Margaret Clagett-Dame (University of Wisconsin-Madison) [4]. To examine the role of GRP75 in neuronal differentiation, SH-SY5Y cells were transiently transfected with the GFP-GRP75 construct or an empty vector (pEGFP-C1) at the indicated concentrations using Lipofectamine 2000 (Invitrogen) according to the manufacturer’s instructions. The shRNA lentiviral vectors targeting human GRP75 (GRP75-sh-1 and GRP75-sh-2) and a GFP-targeting control lentiviral vector (Control-sh) were purchased from the National RNAi Core Facility, Academia Sinica, Taiwan. To generate lentiviral particles, HEK293T cells were co-transfected with one shRNA vector along with two packaging vectors (pMD.G and pCMV∆R8.91) for 48 h. The conditioned media containing recombinant lentiviral particles were harvested and filtered through a 0.45-μM filter. SH-SY5Y cells were transduced with lentiviruses for 48 h to suppress the expression of GRP75.

**Co-immunoprecipitation and Western blotting**

SH-SY5Y cells were treated with 10 μM RA or 0.1% DMSO at 37 °C for 3 d, and the nuclear extracts and cytosolic pools of treated cells were isolated using the Nuclei EZ Prep Kit (Sigma) as described in the manufacturer’s instructions. Patient tumor samples and harvested xenograft tumors were lysed and homogenized in 1 ml lysis buffer (20 mM HEPES, pH 7.4, 2 mM EDTA, 1% Triton X-100, 10% glycerol, 1 mM DTT). Following removal of debris by centrifugation, the protein concentration of clarified lysates was determined using the BCA protein assay kit (Pierce). For co-immunoprecipitation, 20 μg of a mouse anti-GRP75 antibody or a non-specifi mouse IgG was incubated with 200 μl of immobilized protein A agarose in PBS for 2 h at 4 °C. The antibody-conjugated protein A agarose was cross-linked with 1 ml of borate buffer (0.2 M sodium borate, pH 9.0) containing 20 mM DMP for 30 min at room temperature. The reaction was terminated by the addition of 0.2 M ethanolamine (pH 8.0) and incubation for 1 h at room temperature. Following extensive washes with PBS, nuclear extracts or cytosolic pools (1 mg of total proteins) were incubated with antibody-conjugated protein A agarose at 4 °C overnight. Upon the removal of unbound proteins and extensive washes with PBS, immunoprecipitated proteins were eluted by the addition of 4X sample loading buffer and boiling at 100 °C for 10 min, followed by SDS-PAGE and Western blotting. To identify specific proteins in cellular lysates, samples containing equivalent amounts of protein were mixed with 6X sample loading buffer and boiled at 100 °C for 10 min. Proteins were resolved by 10% Tris-glycine polyacrylamide gels and transferred electrophoretically to polyvinylidene difluoride (PVDF) membranes (Pall). Membranes were blocked in 5% BSA in TBST (blocking buffer) for 1 h at room temperature, followed by incubation with the indicated primary antibodies in blocking buffer at 4 °C overnight. Following extensive washes with TBST, membranes were further incubated with HRP-conjugated secondary antibody in blocking buffer for 1 h at room temperature. Following extensive washes with TBST, antibody-reactive proteins were visualized using the Immobilon Western Chemiluminescent HRP Substrate (Millipore). Images were captured and processed with ChemiGenius2 (Syngene).

**Luciferase reporter assay**

SH-SY5Y cells were seeded onto 6-well microplates (5x10^5^ cells/well) and infected with equivalent M.O.I. of shRNAs (control-sh, GRP75-sh-1, or GRP75-sh-2) at 37 °C for 2 d. Following removal of infection mixtures, transduced cells were further transfected with a promoter-specific luciferase reporter gene construct (either RARE-luc, RARb-luc, NEDD9-luc, or MYCN-luc) using Lipofectamine 2000 at 37 °C overnight. Subsequently, transfected cells were treated with 10 μM RA or 0.1% DMSO for 24 h. Promoter-driven luminescence in the clarified lysates of transfected cells was determined using the Steady-Glo luciferase assay reagent (Promega) and normalized to the protein content of the lysates. Normalized luminescence derived from control-sh-infected cells in the presence of 0.1% DMSO is referred to as one fold of relative promoter activity (luciferase activity). The data are shown as the average luciferase activity ± SEM from three independent experiments.

**RNA isolation, reverse transcription, and quantitative real-time PCR**

Total RNA was isolated from transduced SH-SY5Y cells using the TRIzol Reagent (Invitrogen). Purified RNAs (2 μg) were reverse-transcribed to the first-strand cDNA using the SuperScript III First-strand cDNA Synthesis Kit (Invitrogen). Equivalent amounts of cDNA were used in quantitative real-time PCR using the SYBR Green I Master reagent on the LightCycler 480 Real-Time PCR System (Roche) with gene-specific primer pairs. Cycling conditions were 95 °C for 3 min, followed by 40 cycles of 94 °C for 30 s, 55 °C for 30 s, and 72 °C for 1 min. The transcript levels of specific genes were normalized to those of GAPDH. The data were expressed as the average (± SD) of triplicate measurements from three independent experiments. The sequences of primers used in the PCR reaction are listed as follow: GRP75 (Forward), 5´-AGA TAT CTG GAC TGA ATG TGC TTC-3´; GRP75 (Reverse), 5´-ATC ACT TCT CCT ATG TCA CTC TTG-3´; CLMN (Forward), 5´-GTG AAA GAC CAG AGG AAG GCT-3´; CLMN (Reverse), 5´-TGA TGC GAA CAA AAG TGG AT-3´; CRABP2 (Forward), 5´-TCA AAG TGC TGG GGG TGA AT-3´; CRABP2 (Reverse), 5´-TCT GCT CCT CAA ACT CCT CCC-3´; HOXD10 (Forward), 5´-CCC TGA GTC TTG TCC CGT TC-3´; HOXD10 (Reverse), 5´-GGC TCG TTC ATC TTC TTT TCC A-3´; RARβ (Forward), 5´-CCA GGA CAA ATC ATC AGG GT-3´; RARβ (Reverse), 5´-TGG CAG AGT GAA GGG AAA GT-3´; NAV2 (Forward), 5´-CTG TGA CGG AGA GGC TGG AC-3´; NAV2 (Reverse), 5´-TGC TCT TCA TTC CTG GTT TTG G-3´; NEDD9 (Forward), 5´-ATG TCC ACG TCT TCC ACC TCC-3´; NEDD9 (Reverse), 5´-AGT GAC CAG TGC CAT TAG GCT G-3´; RET (Forward), 5´-GGT CTG TGG TGC TGT GGT CT-3´; RET (Reverse), 5´-CAC TCG TCT GTT GTC TGA GC-3´; TH (Forward), 5´-CAG TAC AGG CAC GGC GA-3´; TH (Reverse), 5´-GGA TAT TGT CTT CCC GGT AGC-3´; TrkA (Forward), 5´-CAA CGG CAA CTA CAC GC-3´; TrkA (Reverse), 5´-TGT TTC GTC CTT CTT CTC CAC-3´; GAPDH (Forward), 5´-CAT GTG GGC CAT GAG GTC CAC CAC-3´; GAPDH (Reverse), 5´-GCT TAG CAC CCC TGG CCA AGG TCA-3´.

**Chromatin immunoprecipitation (ChIP)**

SH-SY5Y cells were transduced with shRNA lentivirus (control-sh, GRP75-sh-1, or GRP75-sh-2) for 48 h, followed by treatments with 10 μM RA or 0.1% DMSO and incubation at 37 °C for 24 h. To cross-link histone to DNA, treated cells were fixed with 1% formaldehyde for 10 min at 37 ºC, and the reaction was terminated by the addition of 1.25 M glycine (1:9, v/v). Fixed cells were lysed and sonicated in buffer A (1% SDS, 10 mM EDTA, 50 mM Tris, pH 8.0) to shear DNA. Following the removal of debris by centrifugation, clarified lysates were subject to immunoprecipitation using antibodies (10 μg) against either GRP75, RARα, RXRα, or mouse IgG (as control) at 4 °C overnight with agitation, followed by incubation with protein A-conjugated agarose at 4 °C for 1 h. The DNA/histone/antibody-bound protein A agarose beads were sequentially washed twice for 5 min in buffer B (0.1% SDS, 1% Triton X-100, 2 mM EDTA, 10 mM Tris, pH 8.0, 150 mM NaCl), buffer C (0.1% SDS, 1% Triton X-100, 2 mM EDTA, 10 mM Tris, pH 8.0, 500 mM NaCl), buffer D (0.25 M NaCl, 1% NP-40, 1% sodium deoxycholate, 1 mM EDTA, 10 mM Tris, pH 8.0), and then buffer E (10 mM Tris, pH 8.0, 1 mM EDTA). DNA/histone complexes were eluted twice with buffer F (1% SDS, 0.1 M NaHCO_3_) for 15 min at room temperature with agitation. Eluates were pooled and heated at 65 °C for 4 h. Precipitated DNA fragments were purified using the QIAquick PCR Purification Kit (Qiagen) as described in the manufacturer’s instructions. Input DNA and immunoprecipitated DNA samples were used in the PCR reaction. The sequences of paired primers for the detection of the DR5 RARE within the promoter of *RARβ* were 5´-GGG AGT TTT TAA GCT CTG TGA G-3´ and 5´-TGA ACA GCT CAC TTC CTA CT-3´.

**Protein stability**

SH-SY5Y cells were infected with lentiviral shRNAs for 2 d. Protein synthesis in infected cells was inhibited with 50 μg/ml cycloheximide for 3 h, followed by the addition of 10 μM RA or vehicle alone (0.1% DMSO) and incubation at 37 °C for various intervals. Cells were lysed in lysis buffer (20 mM HEPES, pH 7.4, 2 mM EDTA, 1% Triton X-100, 10% glycerol, 1 mM DTT), and clarified lysates were subjected to immunoblotting using the indicated antibodies.

**Immunohistochemical staining**

The expression of GRP75 in formalin-fixed, paraffin-embedded NB tumor tissues was evaluated by immunohistochemical staining with a goat anti-GRP75 antibody (1:100) as previously described [2]. The nuclei were counterstained with hematoxylin. The specificity of GRP75 immunostaining was confirmed using nonimmunized goat serum and a specific blocking peptide containing the reactive epitope of anti-GRP75 antibody.

**Ubiquitination assay for RARα and RXRα**

SH-SY5Y cells were seeded onto 6-well microplates and infected with GRP75-sh-1, GRP75-sh-2, or control-sh at 37 °C for 2 d, followed by the removal of infection mixture and incubation with fresh medium containing 10 μM RA or 0.1% DMSO for 16 h in the presence or absence of 5 μM MG132. Treated cells were lysed in lysis buffer (20 mM HEPES, pH 7.4, 2 mM EDTA, 1% Triton X-100, 10% glycerol, 1 mM DTT) and subjected to immunoprecipitation with either rabbit anti-RARα or rabbit anti- RXRα antibody as described above. The precipitates were resolved by SDS-PAGE and analyzed by immunoblotting with an anti-ubiquitin antibody.

**Tumor growth in nude mice**

To examine the correlation between the RA-elicited formation of tripartite GRP75/RARα/RXRα complexes and the tumor progression of NB, six-week-old male athymic nude mice were injected s.c. with 1×10^7^ stNB-V1 cells [kindly provided by Dr. Christina Ling Chang, National Cheng Kung University, Tainan, Taiwan [1]] in 500 μl Matrigel (BD Bioscience). When the NB tumor xenografts reached a volume of 1 cm^3^, mice were randomly divided into two groups that were treated intraperitoneally with RA (1 mg/kg b.w.) or vehicle for 2 weeks with daily dosing. At the conclusion of dosing, mice were euthanized and the tumor xenografts were removed to evaluate their weight and volume. Tumor diameters were measured with calipers, and volume was calculated as LxW^2^x0.5, where L and W are the tumor length and width in mm, respectively. This animal study protocol was approved by the Institutional Animal Care and Use Committee of Academia Sinica.

**Patients and sample preparation**

A cohort of 30 histologically confirmed NB patients with complete follow-up protocols approved by the Institutional Review Board of National Taiwan University Hospital, Taipei, Taiwan, were enrolled in this study. Tumor samples were obtained during surgery and immediately frozen in liquid nitrogen. The categorization of tumor biopsies was based on the International Neuroblastoma Pathology Classification scheme [5]. This cohort included eight cases of undifferentiated NB (UNB, Schwannian stroma poor), nine cases of differentiating NB (DNB, Schwannian stroma poor, including poorly differentiated subtype), and thirteen cases of ganglioneuroblastoma, intermixed (GNB, Schwannian stroma-rich). Patients were treated with surgery only or a combination of multiple modalities including chemotherapy, radiotherapy, and/or autologous bone marrow transplantation according to the patient’s risk grouping [6].

**Homology modeling and docking simulation**

The molecular structure of human GRP75 has not been resolved. Among the homologues of GRP75, *Escherichia coli* HSP70 chaperone chain A (PubMed accession P0A6Y8) exhibits the highest amino acid sequence identity to human GRP75. The three-dimensional model of GRP75 was thus generated with the MODELER program encoded in Discovery Studio 2.1 (Accelrys, Inc., CA) using *Escherichia coli* HSP70 chaperone chain A (Protein Data Bank code 2KHO_A) as the template structure. MODELER used a spatial restraint method to build up a 3-D image of the protein structure and was capable of generating a reliable predicted structure using probability density functions derived from homologous structures and general features of known proteins [7]. The coordinates of the higher resolution structure of *E. coli* HSP70 chaperone chain A were used to model the predicted structure of human GRP75 that is chosen with the lowest violation score and lowest energy score. All calculations were carried out in the Discovery Studio 2.1 as previously described [8].

To further explore possible interaction sites between GRP75 and RARα/RXRα heterodimers, the docking of modeled GRP75 structure to known RARα or RXRα structure was simulated using the docking protocol ZDOCK (<http://zdock.bu.edu/>) [9]. The best configuration of a GRP75-RARα or GRP75-RXRα complex was then energy-minimized with CHARMM enabling full flexibility for the ligand and only side-chain flexibility for the receptor [10].

**Statistical analysis**

The band density of the antibody-reactive proteins detected by Western blotting was determined using NIH Image J software. All quantitative results are expressed as the mean ± SEM of three independent experiments. Statistical analyses were performed with a two-tailed Student’s *t*-test. Differences with p values < 0.05 were considered statistically significant. Immunofluorescence signals were quantified by the MetaMorph software (Molecular Device) and analyzed by Student’s *t*-test. Data are shown as the average fluorescence per cell ± SEM from at least three different viewing areas. The number of cells used in quantitative analysis was at least 150 for each group.

**Supplementary References**

1. Almgren MA, Henriksson KC, Fujimoto J, Chang CL (2004) Nucleoside diphosphate kinase A/nm23-H1 promotes metastasis of NB69-derived human neuroblastoma. Mol Cancer Res 2: 387-394.

2. Hsu WM, Lee H, Juan HF, Shih YY, Wang BJ, et al. (2008) Identification of GRP75 as an independent favorable prognostic marker of neuroblastoma by a proteomics analysis. Clin Cancer Res 14: 6237-6245.

3. Srinivas H, Xia D, Moore NL, Uray IP, Kim H, et al. (2006) Akt phosphorylates and suppresses the transactivation of retinoic acid receptor alpha. Biochem J 395: 653-662.

4. Knutson DC, Clagett-Dame M (2008) atRA Regulation of NEDD9, a gene involved in neurite outgrowth and cell adhesion. Arch Biochem Biophys 477: 163-174.

5. Shimada H (2003) The International Neuroblastoma Pathology Classification. Pathologica 95: 240-241.

6. Cohn SL, Pearson AD, London WB, Monclair T, Ambros PF, et al. (2009) The International Neuroblastoma Risk Group (INRG) classification system: an INRG Task Force report. J Clin Oncol 27: 289-297.

7. Fiser A, Sali A (2003) Modeller: generation and refinement of homology-based protein structure models. Methods Enzymol 374: 461-491.

8. Huang TC, Huang HC, Chang CC, Chang HY, Ou CH, et al. (2007) An apoptosis-related gene network induced by novel compound-cRGD in human breast cancer cells. FEBS Lett 581: 3517-3522.

9. Chen R, Weng Z (2003) A novel shape complementarity scoring function for protein-protein docking. Proteins 51: 397-408.

10. Brooks BR, Brooks CL, 3rd, Mackerell AD, Jr., Nilsson L, Petrella RJ, et al. (2009) CHARMM: the biomolecular simulation program. J Comput Chem 30: 1545-1614.
